# Improved biomarker discovery through a plot twist in transcriptomic data analysis

**DOI:** 10.1186/s12915-022-01398-w

**Published:** 2022-09-24

**Authors:** Núria Sánchez-Baizán, Laia Ribas, Francesc Piferrer

**Affiliations:** Institut de Ciències del Mar (ICM), Spanish National Research Council (CSIC), Barcelona, Passeig Marítim, 37-49, 08003 Barcelona, Spain

**Keywords:** Gene expression analysis, Gene networks, Weighted gene co-expression network analysis (WGCNA), Sex determination and differentiation, Gonadal development, Biomarker discovery

## Abstract

**Background:**

Transcriptomic analysis is crucial for understanding the functional elements of the genome, with the classic method consisting of screening transcriptomics datasets for differentially expressed genes (DEGs). Additionally, since 2005, weighted gene co-expression network analysis (WGCNA) has emerged as a powerful method to explore relationships between genes. However, an approach combining both methods, i.e., filtering the transcriptome dataset by DEGs or other criteria, followed by WGCNA (DEGs + WGCNA), has become common. This is of concern because such approach can affect the resulting underlying architecture of the network under analysis and lead to wrong conclusions. Here, we explore a plot twist to transcriptome data analysis: applying WGCNA to exploit entire datasets without affecting the topology of the network, followed with the strength and relative simplicity of DEG analysis (WGCNA + DEGs). We tested WGCNA + DEGs against DEGs + WGCNA to publicly available transcriptomics data in one of the most transcriptomically complex tissues and delicate processes: vertebrate gonads undergoing sex differentiation. We further validate the general applicability of our approach through analysis of datasets from three distinct model systems: European sea bass, mouse, and human.

**Results:**

In all cases, WGCNA + DEGs clearly outperformed DEGs + WGCNA. First, the network model fit and node connectivity measures and other network statistics improved. The gene lists filtered by each method were different, the number of modules associated with the trait of interest and key genes retained increased, and GO terms of biological processes provided a more nuanced representation of the biological question under consideration. Lastly, WGCNA + DEGs facilitated biomarker discovery.

**Conclusions:**

We propose that building a co-expression network from an entire dataset, and only thereafter filtering by DEGs, should be the method to use in transcriptomic studies, regardless of biological system, species, or question being considered.

**Supplementary Information:**

The online version contains supplementary material available at 10.1186/s12915-022-01398-w.

## Background

The transcriptome is defined as the entire set of messenger RNA (mRNA or transcripts) expressed by a cell or tissue type of an organism of a given genotype under certain internal and external influences. The total amount and types of transcripts vary depending on multiple factors such as stage of development, physiological state, or environmental conditions [1]. Transcriptomics, the analysis of the whole transcriptome, started using complementary DNA (cDNA) clones to generate expressed sequence tags (ESTs), and the development of the first microarrays in the early 90s [2–4]. An accurate comprehension of the transcriptome is essential to reveal the molecular constituents of cells and tissues, to understand the functional elements of the genome, and also to gain a better understanding of development and disease [5]. With the advent of high-throughput technologies to analyze the transcriptome, several bioinformatic challenges appeared in storing, pre-processing, and analyzing the large datasets produced. As a consequence, a variety of bioinformatic pipelines arose to overcome such challenges [6, 7]. To date, the most commonly used method in various fields of biology and medicine is screening for differentially expressed genes (DEGs), which compares the mean expression levels for individual genes between two or more groups of samples [8–15].

In 2005, Zhang and Horvath proposed a bioinformatic application called weighted gene co-expression network analysis (WGCNA) to gain a deeper understanding of the transcriptome and to elucidate the underlying cellular processes based on the coordinated co-expression of genes encoding the interacting proteins [16]. In contrast to DEG analysis, WGCNA is a gene screening method that takes advantage of the inherent variability in gene expression among different biological samples to illuminate higher-order relationships among genes. WGCNA generates clusters (called modules) of strongly correlated genes based on the Pearson correlation as a measure of their functional relatedness and assigns a different color to each module for easy identification purposes. WGCNA is a powerful method and has many advantages over DEG analysis since it also allows evaluating the association of modules with phenotypic sample traits using network properties. Furthermore, WGCNA facilitates the identification of candidate biomarkers and hub genes relevant to the process under study. The weighted co-expression network consists of an adjacency matrix reporting the connection strength between gene pairs [16, 17]. Since its development, its use has become exponentially widespread and allows integrating network parameters with genetic information from microarray datasets and, more recently, from RNA sequencing experiments [18–20]. It should be noted that variations or methods other than WGCNA have also been developed, such as Differential Co-expression Analysis or metaDCN [21], THD-Module Extractor [22], Diffcoex [23], and module differential analysis for weighted gene co-expression network (MODA) [24]. However, WGCNA remains by far the most commonly used in numerous research fields.

**Fig. 1:**
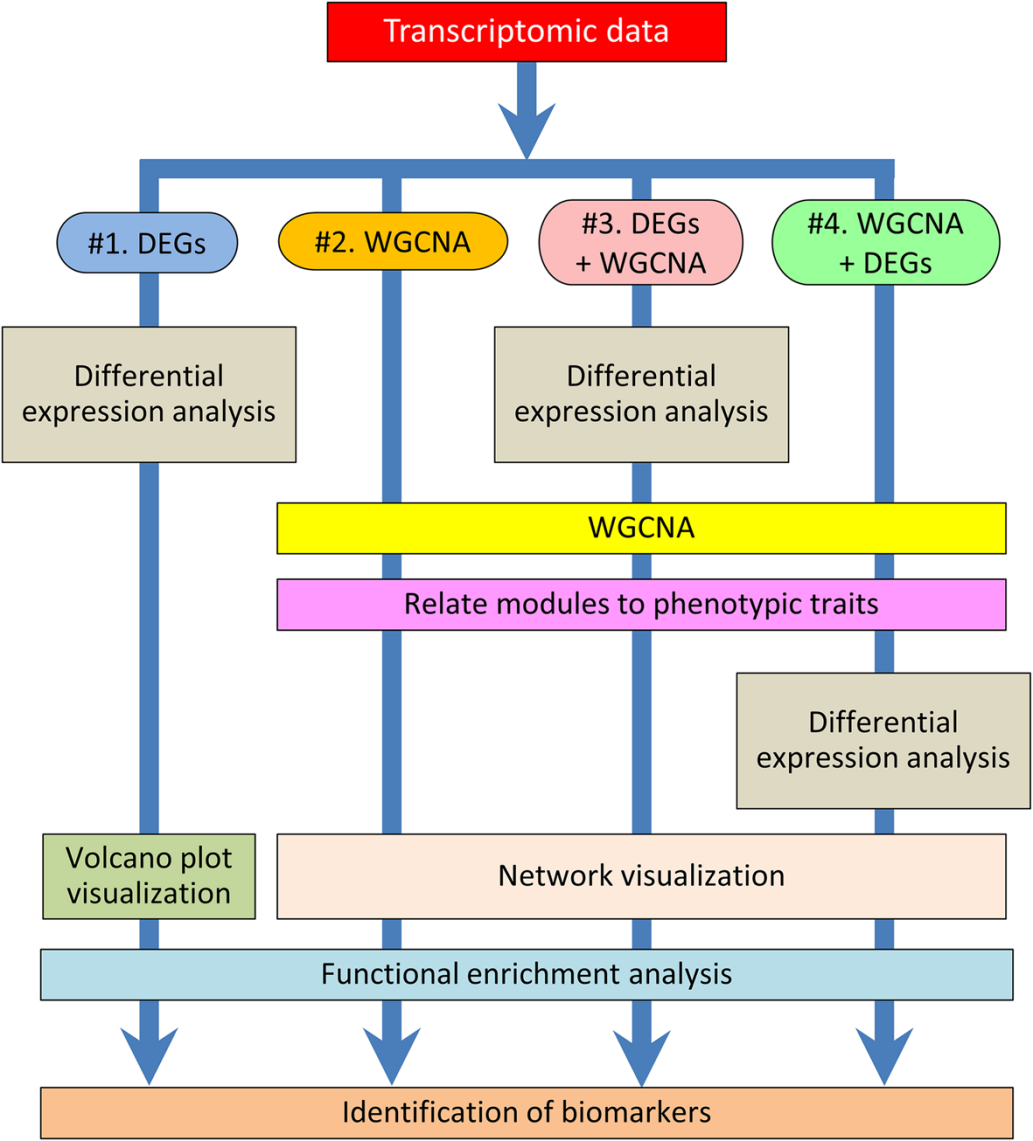
Flow diagram of the three most used methods in the literature to analyze transcriptomic data plus one method (#4) proposed. Method #1 Analysis of the differentially expressed genes (DEGs) between two conditions followed by volcano plot visualization, functional enrichment analysis (FEA), and biomarker identification. #2 Construction of a gene co-expression network by using the WGCNA package (Langfelder and Horvath, [17]) in R, following the general WGCNA guidelines to perform FEA, identify modules and genes related to a trait of interest (Zhang and Horvath, 2005). #3 To filter the transcriptome by DEGs and conduct WGCNA using the filtered dataset and continue with FEA. In this study, we propose method #4 to use WGCNA and filter the output of the selected modules by DEGs to follow with FEA and biomarker discovery

In reviewing the literature extensively, we found three main strategies to analyze the transcriptome (Fig. 1) that can be broadly classified as (1) screening for differentially expressed genes between two conditions (from now on referred to as method #1 or simply as DEGs), (2) WGCNA and derivatives (from now on referred as method #2 or simply as WGCNA), and (3) a method that has become also very popular that can be viewed as a combination of the two former and consists in filtering the transcriptome dataset by DEGs or other criteria, e.g., considering only the most expressed genes, or the top 25% genes with more expression variance, etc., and only then applying WGCNA to the filtered dataset (from now on referred to as method #3 or DEGs + WGCNA). Another strategy that we have found in reviewing the literature is the independent use of methods #1 (DEGs) and #2 (WGCNA) in the same study but then using only one of the two for the subsequent downstream analysis on the data [13, 25–32]. Some examples of studies following each one of the three broad methods defined above are shown in Table 1. Of note, regardless of the method used, usually, these analyses are followed by data visualization and functional analysis (gene ontology and/or pathways enrichment analysis).

**Table 1:** Examples of some transcriptomic studies classified according to the three different methods described (#1, DEGs; #2, WGCNA; # 3, DEGs + WGNCA; # 1 + 2 indicates the use of both methods, without combining them, as in #3) to analyze transcriptomic data since the development of WGCNA in 2005

Method #	Species	Cell type/ tissue	Technology	Reference
1	*Rattus norvegicus*	Gonads	Microarray	[9]
1	*Setaria italica L*	Plant seeds	SSH	[11]
1	*Gallus gallus*	Gonads	RNA-seq	[33]
1	*Trachemys scripta*	Embryos	RNA-seq	[15]
1	*Triticum aestivum L*	Plant seeds	Microarray	[14]
1	*Homo sapiens*	Breast	RNA-seq	[12]
1	*Xenopus laevis*	Gonads	Microarray	[34]
1	*Rattus norvegicus*	Brain	RNA-seq	[10]
1	*Dicentrarchus labrax*	Gonads	Microarray	[35]
2	*Homo sapiens*	Brain	Microarray	[36]
2	*Homo sapiens*	Brain	Microarray	[37]
2	*Homo sapiens*	Brain	RNA-seq	[19]
2	*Homo sapiens*	Bone	Microarray	[38]
2	*Homo sapiens*	Liver	Microarray	[39]
2	*Mus musculus*	Placenta	Microarray	[40]
2	*Homo sapiens*	Brain	Microarray	[41]
2	*Oplegnathus fasciatus*	Spleen	RNA-seq	[42]
2	*Homo sapiens*	Blood	RNA-seq	[43]
2	*Homo sapiens*	Lung	Microarray	[44]
2	*Homo sapiens*	Bladder	RNA-seq	[20]
3	*Homo sapiens*	Bladder	Microarray	[45]
3	*Homo sapiens*	Colon mucose	Microarray	[46]
3	*Homo sapiens*	Podocyte cells	Microarray	[47]
3	*Bubalus bubalis*	Mammary gland	RNA-seq	[48]
3	*Homo sapiens*	Bladder	Microarray	[49]
3	*Homo sapiens*	Lung	Microarray	[50]
3	*Homo sapiens*	Bladder	RNA-seq	[51]
3	*Bubalus bubalis*	Blood	RNA-seq	[52]
3	*Homo sapiens*	Brain	RNA -seq	[53]
3	*Homo sapiens*	Liver	RNA-seq	[54]
3	*Scophthalmus maximus*	Gill	RNA-seq	[55]
3	*Scophthalmus maximus*	Kidney	RNA-seq	[56]
3	*Homo sapiens*	Breast	Microarray	[57]
3	*Rattus norvegicus*	Spinal cord	RNA-seq	[58]
3	*Homo sapiens*	Stem cells	RNA-seq	[59]
3	*Bos taurus*	Blood	RNA-seq	[60]
1 + 2	*Scophthalmus maximus*	Gonads	Microarray	[13]
1 + 2	*Paralichthys olivaceus*	Embryo	RNA-seq	[25]
1 + 2	*Homo sapiens*	Brain	RNA-seq	[26]
1 + 2	*Gallus gallus*	Lung	RNA-seq	[27]
1 + 2	*Homo sapiens*	Organoids	RNA-seq	[28]
1 + 2	*Homo sapiens*	Placenta	Microarray	[29]
1 + 2	*Mus musculus*	Gonads	RNA-seq	[30]
1 + 2	*Homo sapiens*	Lung	RNA-seq	[31]
1 + 2	*Mus musculus*	Pancreas	Microarray	[32]

The emergence of method #3 (DEGs + WGCNA) apparently seems a logical forward step to take: by first filtering DEGs, the analysis is limited to a set of previously selected genes, based on a statistical or quantitative threshold and, thus, it requires less computational power than the required by WGCNA of the entire dataset. However, and this is very important for the proper analysis of gene interactions, in a scale-free network (i.e., a network whose characteristics are independent of the size or number of nodes making up the network) such as the gene networks, the fraction of nodes with degree *k*, where *k* is connectivity (i.e., the sum of connections of a node), follows a power-law *k*^−*α*^ distribution, where *α* is some exponential. In this manner, the network topology is dominated by a few highly connected nodes, called hubs, linked to the rest of less connected nodes [16]. Therefore, applying a filtering step such as DEGs previous to WGCNA, as done in method #3, might eliminate many of the less connected genes that, if not filtered, would contribute to some nodes being identified as hubs. This is of great concern because it thus could affect the core architecture of the network and lead to biased results and interpretations. In contrast, construction of a network with the unfiltered dataset allows drawing the complete map of the network.

As we have just seen, method #3 has the main advantage that one ends up dealing with a much-reduced gene list, albeit all genes are DEGs, but it has serious dangers that can affect the architecture of the networks and lead to false conclusions. Furthermore, the validity of method #3 was never established and, in fact, the developers of method #2 (WGCNA) recommend against the application of such filter before WGCNA [61]. Thus, to combine the benefits from WGCNA, taking advantage of the whole dataset and not affecting the topology of the network, with the strength and relative simplicity of DEG analysis, in the present study we propose a sort of plot twist in transcriptome data analysis. We propose a new strategy (referred to as method #4): to perform WGCNA using the entire transcriptome dataset and, only subsequently, filtering by DEGs (Fig. 1). To validate this new method, we compared it with the performance of method #3, which is, as mentioned above, nowadays much spread in the literature.

For the purpose of this study, we selected one of the most transcriptomically complex tissues, the gonads [62] and one of the most complicated developmental processes that involve many genes interacting in a delicate spatio-temporal and orchestrated manner: vertebrate gonadal sex differentiation. At the bi-potential stage, the vertebrate gonad expresses genes from the pro-male (leading to testis differentiation) and pro-female (leading to ovarian differentiation) pathways with opposing effects until the time when, depending on the species, a combination of genetic and/or environmental influences switch the balance towards the differentiation of one sexual phenotype and the repression of the other [63]. Thus, vertebrate gonadal sex differentiation provides an excellent opportunity to compare different methods to analyze large amounts of gene expression data. Furthermore, we used data from the European sea bass, *Dicentrarchus labrax* (from now on sea bass), a modern teleost with a polygenic sex-determining (PSD) mechanism without sex chromosomes [64], the mouse model, where gonadal sex differentiation has been extensively studied, and the human, a species where despite the limited availability of normal fetal gonads, it has been extensively studied for better understanding of the complex pathways related to disorders of sexual development (DSDs) [65, 66]. As with most mammals, mouse and human have an XX/XY sex-determining system where SRY is the master male sex-determining gene [67]. These three species with different sex-determining mechanism and differentiation dynamics were used to show that our approach was not only working on a complex process but also to show that the results are consistent regardless of the underlying genetic architecture. In all cases, we show that method #4 provides a more realistic and nuanced picture of the complex gene interactions taking place over time during gonadal sex differentiation and propose this method for future transcriptomic studies regardless of the biological system or question being considered.

## Results

### Construction of WGCNA using different approaches

WGCNA was conducted on male and female samples at two developmental stages (sea bass and mouse) or three developmental stages (human) to investigate the gene network operating during gonadal development in the sea bass, mouse, and human (Supplementary Figure 1).

**Fig. 2:**
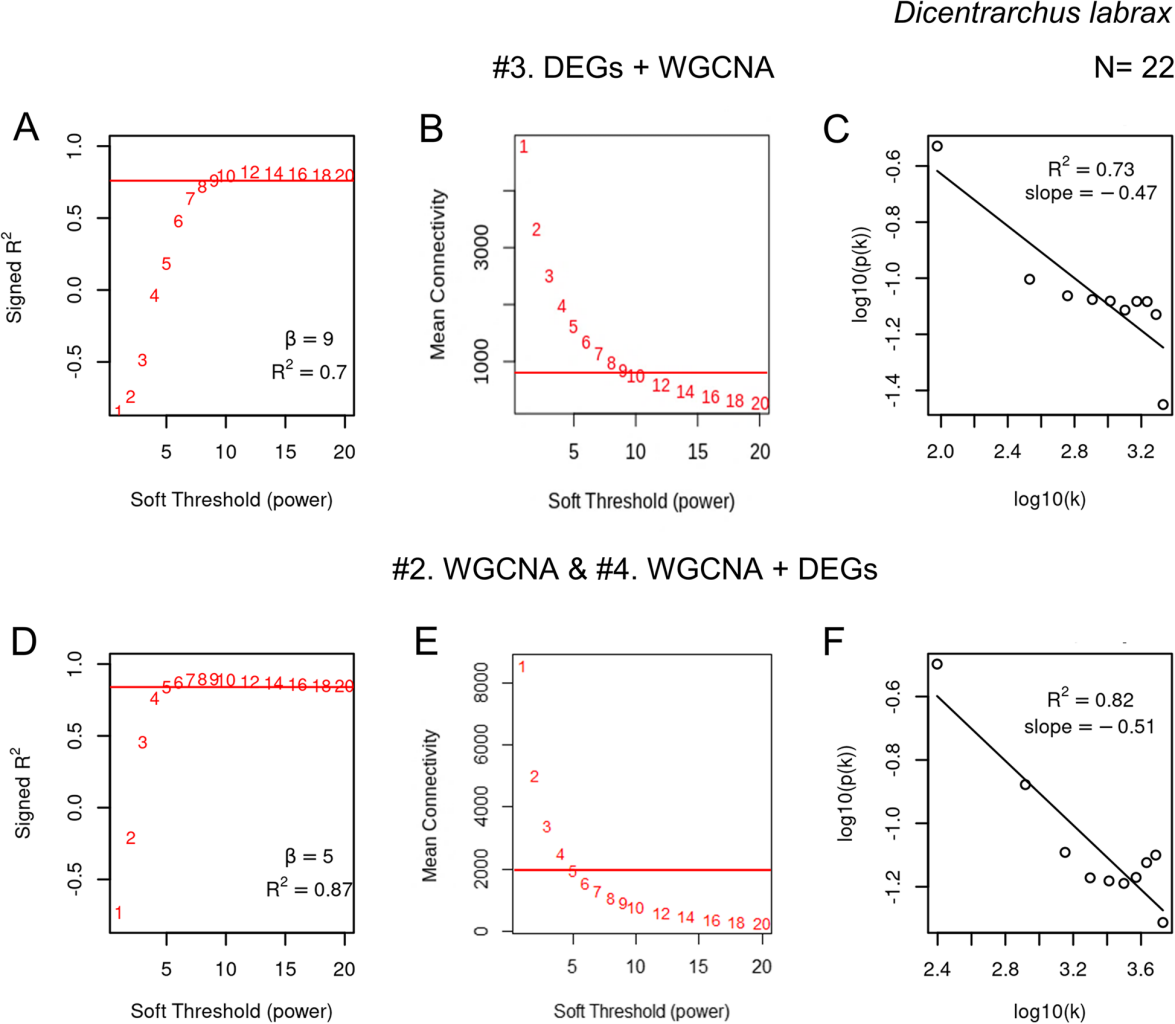
WGCNA model fit and soft threshold determination. Soft thresholding power analysis was used to obtain the scale-free fit index (ranging from 1 to 20) of network topology, for method #3 (**A**) and #2 and #4 (**D**), which until that step of the working pipeline are the same. Mean connectivity when using method #3 (**B**) and method #4 (**E**). The plot of log10(*p*(*k*)) vs log10(*k*) indicates that by using the chosen *β* value, **C** method #3 (*β* = 9) and **F** method #4 (*β* = 5), the network is close to a scale-free network because it is approximately following a straight line. *k* is the whole network connectivity and *p*(*k*) is the corresponding frequency distribution

We applied method #3 to the sea bass transcriptome, composed of a total of 20,978 genes, and identified 8434 DEGs between males and females at 250 days post fertilization (dpf) (*P* < 0.05) (Supplementary Figure 2). The normalized intensities of the 8434 DEGs (Additional file 1) were then used for WGCNA to build the network. The selected soft threshold used for the adjacency function closest to meet scale-free topology criterion and additional considerations was *β* = 9, leading to signed *R*^2^ = 0.7 (Fig. 2A). Regarding connectivity, the second parameter considered for the selection of the soft threshold indices, the mean value was < 1000 (Fig. 2B) while the slope of the regression line between log10 (*p*(*k*)) and log10 (*k*) was − 0.47 (*R*^2^ = 0.73) (Fig. 2C). In contrast, when the entire transcriptome was used (methods #2. WGCNA and #4. WGCNA + DEGs), the selected soft threshold obtained was *β* = 5, which resulted in a better fit, signed *R*^2^ = 0.87 (Fig. 2D), and more than doubled the mean connectivity to ~ 2000 (Fig. 2E) while also improving the slope − *γ* =  − 0.51 (*R*^2^ = 0.82) (Fig. 2F).

The same difference between the two methods was observed in the mouse. Analysis of the gonadal transcriptome between males and females at 16.5 days post coitum (dpc) (*P* < 0.05) identified 8109 DEGs (Additional file 2). Using method #3, the selected soft threshold was *β* = 14 which resulted in a signed *R*^2^ = 0.55 (Supplementary Figure 3 A), a mean connectivity value < 500 (Supplementary Figure 3 B), and slope − *γ* =  − 0.79 (*R*^2^ = 0.51) (Supplementary Figure 3 C). However, when the entire gene expression dataset (14,088 genes) were used for the construction of the network (methods #2. WGCNA and #4. WGCNA + DEGs), the soft threshold was *β* = 12, resulting in signed *R*^2^ = 0.7 (Supplementary Figure 3 D), the mean connectivity ~ 500 (Supplementary Figure 3 E), and the slope improved to − *γ* =  − 0.88 (*R*^2^ = 0.55) (Supplementary Figure 3 F).

The discrepancies between the methods were replicated using human data as well, where using method #3 the selected soft threshold was *β* = 8, resulting in signed *R*^2^ = 0.67 (Supplementary Figure 4 A), a mean connectivity value < 50 (Supplementary Figure 4 B), and slope − *γ* =  − 1.02 (*R*^2^ = 0.63) (Supplementary Figure 4 C). With methods #2 WGCNA and #4 WGCNA + DEGs, the soft threshold was *β* = 8, resulting in signed *R*^2^ = 0.89 (Supplementary Figure 4 D), the mean connectivity > 200 (Supplementary Figure 4 E), and the slope improved to − *γ* =  − 1.38 (*R*^2^ = 0.87) (Supplementary Figure 4 F).

To ensure that the improvement in the model fit were not caused by the selection of a particular soft threshold, results were calculated for a range of thresholds (Table 2). In the three species, method #4 outperformed method #3 when it referred to scale-free topology model fit, mean connectivity, and the slope of the regression line between log10 (*p*(*k*)) and log10 (*k*) being closer to − 1, regardless of the soft threshold selected.

**Table 2:** Comparison of model fit parameters under different soft thresholds using methods #3 (DEGs + WGCNA) and #4 (WGCNA + DEGs) on the gonadal transcriptome during early sex differentiation in three different species: sea bass, mouse, and human. The selected threshold and results obtained with the selected threshold are shown in bold

	**Method**	**Threshold**	**Signed *R***^**2**^** (model fit)**	**Mean connectivity**	**Slope**	***R***^**2**^
					**(scale-free topology)**	
*Dicentrarchus labrax*	#3 DEGs + WGCNA	5	0.19	1580	− 0.12	0.08
		7	0.63	1100	− 0.33	0.59
		**9**	**0.7**	**800**	− **0.47**	**0.73**
		11	0.8	650	− 0.56	0.79
		13	0.8	450	− 0.64	0.81
	#4 WGCNA + DEGs	**5**	**0.89**	**2050**	− **0.51**	**0.82**
		7	0.9	1400	− 0.63	0.87
		9	0.91	900	− 0.71	0.88
		11	0.9	700	− 0.78	0.87
		13	0.9	500	− 0.83	0.86
*Mus musculus*	#3 DEGs + WGCNA	8	0.13	540	0.02	− 0.3
		10	0.33	375	− 0.5	0.24
		12	0.47	300	− 0.65	0.4
		**14**	**0.55**	**230**	− **0.79**	**0.51**
		16	0.62	180	− 0.89	0.57
	#4 WGCNA + DEGs	8	0.62	495	− 0.62	0.4
		10	0.67	325	− 0.77	0.49
		**12**	**0.7**	**220**	− **0.88**	**0.55**
		14	0.7	200	− 0.96	0.59
		16	0.73	160	− 1.02	0.62
*Homo sapiens*	#3 DEGs + WGCNA	4	0.45	105	− 0.74	0.38
		6	0.61	50	− 0.93	0.56
		**8**	**0.67**	**25**	− **1.02**	**0.63**
		10	0.68	19	− 1.1	0.64
		12	0.69	15	− 1.17	0.65
	#4 WGCNA + DEGs	4	0.7	950	− 1.3	0.66
		6	0.79	410	− 1.36	0.77
		**8**	**0.89**	**210**	− **1.38**	**0.87**
		10	0.93	100	− 1.42	0.92
		12	0.95	85	− 1.45	0.95

### Selection of modules related to sex

**Fig. 3:**
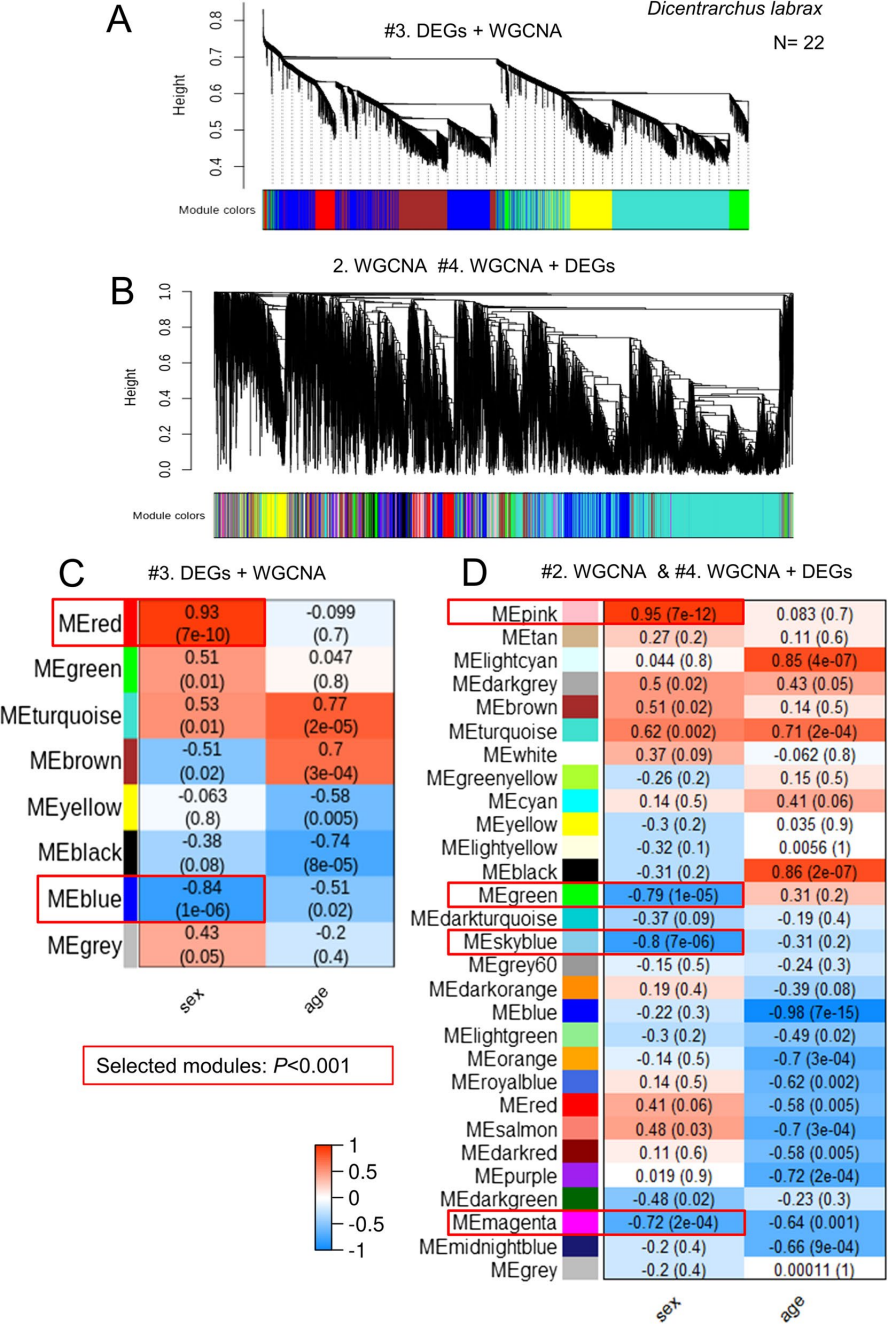
Identification of gene modules associated with sex. Gene hierarchical cluster analysis using method #3 (**A**) and #4 (**B**) using the sea bass gonadal transcriptome. Heat map of the correlation of sex with module eigengene distances using method #3 (**C**) and #4 (**D**). Each color represents a module in the constructed gene co-expression network by WGCNA. The heat map is colored from red (1, positive) to blue (− 1, negative) to indicate the level of correlation of each module with the trait of interest. The red boxes highlight the selected modules for further analysis associated with sex (*P* < 0.01) (color in the online version). Genes not assigned to any of the previous modules are included in the gray module

Because method #3 uses a smaller dataset than method #4, a different number of modules were produced using the average linkage hierarchical clustering algorithm in WGCNA. Thus, in the sea bass the 8434 DEGs retained in method #3 were grouped into eight modules, of which two were strongly associated with the trait of interest, sex (red module: *R*^2^ = 0.93, *P* = 7e − 10; blue module: *R*^2^ =  − 0.84, *P* = 1e − 06) (Fig. 3A, C). On the other hand, the 20,978 genes of methods #2 and #4 were grouped into 29 modules, of which four were strongly associated with sex (pink, *R*^2^ = 0.95, *P* = 7e − 12; green, *R*^2^ =  − 0.79, *P* = 1e − 05; blue sky, *R*^2^ =  − 0.8, *P* = 7e − 06; magenta *R*^2^ =  − 0.72, *P* = 2e − 04) (Fig. 3B, D).

In the mouse, the 8109 DEGs retained in method #3 were clustered into six modules, of which two were associated with sex (blue: *R*^2^ = 0.69, *P* = 0.01; turquoise: *R*^2^ =  − 0.8, *P* = 0.002). Using the entire transcriptome in methods #2 and #4, a total of 21 modules were obtained, of which two were associated with sex (red: *R*^2^ = 0.98, *P* = 1e − 07; brown: *R*^2^ =  − 0.75, *P* = 0.008) (Supplementary Figure 5).

The 1479 differentially expressed transcripts at 6 postconceptional weeks (PCW) in human were grouped into eight modules, among which three of them were positively associated with sex (red: *R*^2^ = 0.64, *P* = 9e − 05; blue: *R*^2^ = 0.8, *P* = 3e − 08; yellow: *R*^2^ = 0.87, *P* = 2e − 10), while one module was negatively associated with sex (turquoise: *R*^2^ =  − 0.91, *P* = 8e − 13). In contrast, the analysis of 35,194 transcripts using method #4 resulted in 36 modules, of which seven modules were associated with sex (light-yellow: *R*^2^ =  − 0.6, *P* = 3e − 04; black: *R*^2^ =  − 0.85, *P* = 5e − 10; dark olive-green: *R*^2^ =  − 0.57, *P* = 6e − 04; red: *R*^2^ =  − 0.72, *P* = 4e − 06; dark red: *R*^2^ =  − 0.66, *P* = 3e − 05; green: *R*^2^ = 0.72, *P* = 4e − 06; green-yellow: *R*^2^ = 0.78, *P* = 2e − 07) (Supplementary Figure 6).

**Fig. 4:**
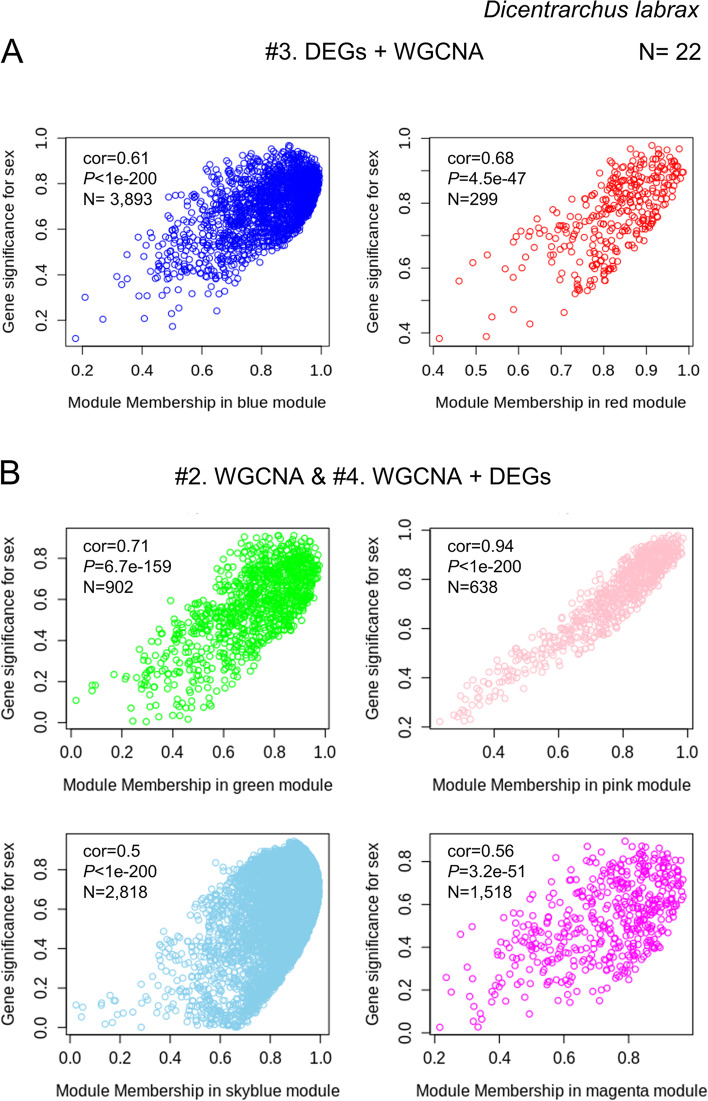
Filtering of genes based on network properties in the sea bass. Scatterplots of correlation between GS vs module membership of each module which were used for determination of interesting modules for sex trait in the gonadal transcriptome of sea bass using method #3 (**A**) and #2 and/or #4 (**B**). The modules were selected when GS was positively and significantly correlated to modular membership. Gene significance is defined as − log of the *p*-value of association of the gene with the trait. Module membership (*k*_ME_) measures how correlated each gene is to a particular module eigengene [68]

Next, considering only the genes from the modules that significantly associated with sex, we selected those that, in addition, showed a significant correlation between modular membership and the gene significance for sex. All the modules associated with sex in the sea bass using methods #3 (Fig. 4A), #2, and #4 (Fig. 4B) showed a significant positive correlation and were kept for further analysis. However, in the weighted network obtained from the mouse data using method #3 the blue module was discarded because it did not pass the established criteria (cor = 0.25, *P* = 1.8e − 28), while the turquoise module was kept for further analysis (cor = 0.64, *P* < 1e − 200) (Supplementary Figure 7 A). Also the two modules associated with sex obtained from mouse data using the methods #2 (WGCNA) and #4 (WGCNA + DEGs) were kept for further analysis given their positive significant correlation (red: 0.96, *P* < 1e − 200; brown: 0.67, *P* < 1e − 200) (Supplementary Figure 7 B). In human, four modules were retained using method #3 (red: 0.75, *P* = 5.3e − 99; blue: 0.81, *P* = 4.6e − 120; yellow: 0.87, *P* = 6.6e − 27; turquoise: 0.84, *P* = 1.7e − 145) (Supplementary Figure 8 A), and seven modules were kept for further analysis using method #4 (light-yellow: 0.74, *P* < 1e − 200; black; 0.86, *P* < 1e − 200; dark green: 0.8, *P* = 1.4e − 13; red: 0.77, *P* < 1e − 200; dark red; 0.81, *P* = 1.3e − 56, green: 0.74, *P* < 1e − 200; yellow-green: 0.82, *P* < 1e − 200) (Supplementary Figure 8 B). Thus, a total of 5876 genes from the four selected modules in sea bass, 3069 genes obtained from the three chosen modules in the mouse, and 8452 transcripts within the seven selected modules from human were kept for further analysis with method #4 (Supplementary Figure 2).

### Co-expressed DEGs involved in gonadal differentiation

After the selection of interesting modules, we further filtered the genes within those modules by its gene significance (GS) for sex (|GS|> 0.2) and intra-modular membership (|*k*_IM_|> 0.8). Furthermore, to implement the method #4 (WGCNA + DEGs), we further filtered the gene lists with the DEGs between males and females at the second stage of development, i.e., 250 dpf in the sea bass, 16.5 dpc in the mouse, and 6 PCW in the human, to identify the most relevant genes of each module. The number of genes co-expressed and, further, that are DEGs at 250 dpf was 3782 in sea bass (pink modules, *n* = 365; green module, *n* = 621; magenta module, *n* = 930; sky blue module, *n* = 1866 genes) (Additional file 3 and Supplementary Figure 2). In the mouse, there were 2205 co-expressed DEGs at 16.5 dpc (red module, *n* = 544; brown module, *n* = 1661) (Additional file 4), and in the human there were a total of 1023 differentially co-expressed transcripts (Supplementary Figure 2).

### Comparison of methods using network properties and statistics

We calculated several topological parameters to compare the performance of methods #2 (WGCNA), #3 (DEGs + WGCNA), and #4 (WGCNA + DEGs). Some of the most relevant parameters are shown in Table 3, including (1) the number of nodes, (2) number of edges, (3) characteristic path length, (4) average number of neighbors, (5) heterogeneity, (6) betweenness centrality, (7) mean degree, and (8) maximum degree. The detailed definitions of topological parameters were published by the developer of the *NetworkAnalyzer* [69]. In the three species, method #4 showed a lower number of nodes than method #2 since only co-DEGs were retained; however, method #4 resulted in a much higher number of nodes and edges than method #3 in the sea bass and human. The average number of neighbors, which indicates the average connectivity of a node, showed higher (better) results when using method #4 than method #3, in proportion to the number of nodes obtained from each method (average number of nodes/ number of nodes) × 100, in sea bass: method #2 = 75%, method #3 = 14%, method #4 = 72%, and mouse: method #2 = 40%, method #3 = 10%, method #4 = 58%, but not in human: method #2 = 3.3%, method #3 = 37%, method #4 = 4.2%. Regarding the characteristic path length, the expected distance between two connected nodes (the shortest, the more compacted is a network), method #4 showed better results than method #3, except in human where the network of the module from method #3 was much smaller and, hence, resulted in shorter characteristic path length. Network heterogeneity is an important parameter for biomarker discovery as it reflects the tendency of a network to contain hub nodes [70]. Such parameter was higher when using method #4 in the sea bass and the human. The maximum betweenness centrality was obtained from method #3 in the sea bass and the mouse, as well as from the human using method #4. The maximum degree was higher in the three species when using method #4. Taken together, the topological parameters of the different networks produced using the three different methods showed that the most robust networks were achieved using methods #2 and #4. Besides, method #4 allows further filtering of the interesting genes without altering main network properties and without removing too many genes that could be importantly related to the trait of interest. Of note, when method #3 resulted in better parameter values, this was always in the mouse, incidentally the species in which the minimum number of samples recommended for network construction was not reached (*n* < 15), leading to less robust conclusions. Considering this and that the construction of the network in the mouse required the highest soft threshold (*β* = 12 in the mouse vs *β* = 5 in the sea bass and *β* = 8 in the human) while achieving the poorer model fit using method #4, the results support the original recommendation of at least 15 samples to construct networks with method #2 (WGCNA) and #4 (WGCNA + DEGs).

**Table 3:** Network statistics results when using methods #2, #3, and #4 in sea bass, mouse, and human transcriptomic data

	**Network statistics**	**Method #2** **WGCNA**	**Method #3** **DEGs + WGCNA**	**Method #4** **WGCNA + DEGs**
*Dicentrarchus labrax*	No. of nodes	638	291	365
	No. of edges	180,759	6059	98,783
	Average no. of neighbors	480.7	42.3	263.42
	Characteristic path length	1.37	2.35	1.66
	Network heterogeneity	0.34	0.68	0.77
	Mean degree	480.7	41.6	263.42
	Max degree	720	135	349
	Max betweenness	0.003	0.07	0.03
*Mus musculus*	No. of nodes	2257	2697	1661
	No. of edges	1,016,156	377,169	798,757
	Average no. of neighbors	903,648	280.21	964.1
	Characteristic path length	1.66	2.39	1.43
	Network heterogeneity	0.563	0.865	0.365
	Mean degree	900.4	105.2	964.1
	Max degree	1867	327	1556
	Max betweenness	0.003	0.02	0.002
*Homo sapiens*	No. of nodes	558	48	481
	No. of edges	5632	382	3375
	Average no. of neighbors	20.2	15.9	14.0
	Characteristic path length	2.4	1.7	2.2
	Network heterogeneity	1.88	0.72	2.3
	Mean degree	20.2	15.9	14.03
	Max degree	309	42	309
	Max betweenness	0.25	0.09	0.39

### Proportion of genes related to sexual development identified by the different approaches in the sea bass and the mouse

**Fig. 5:**
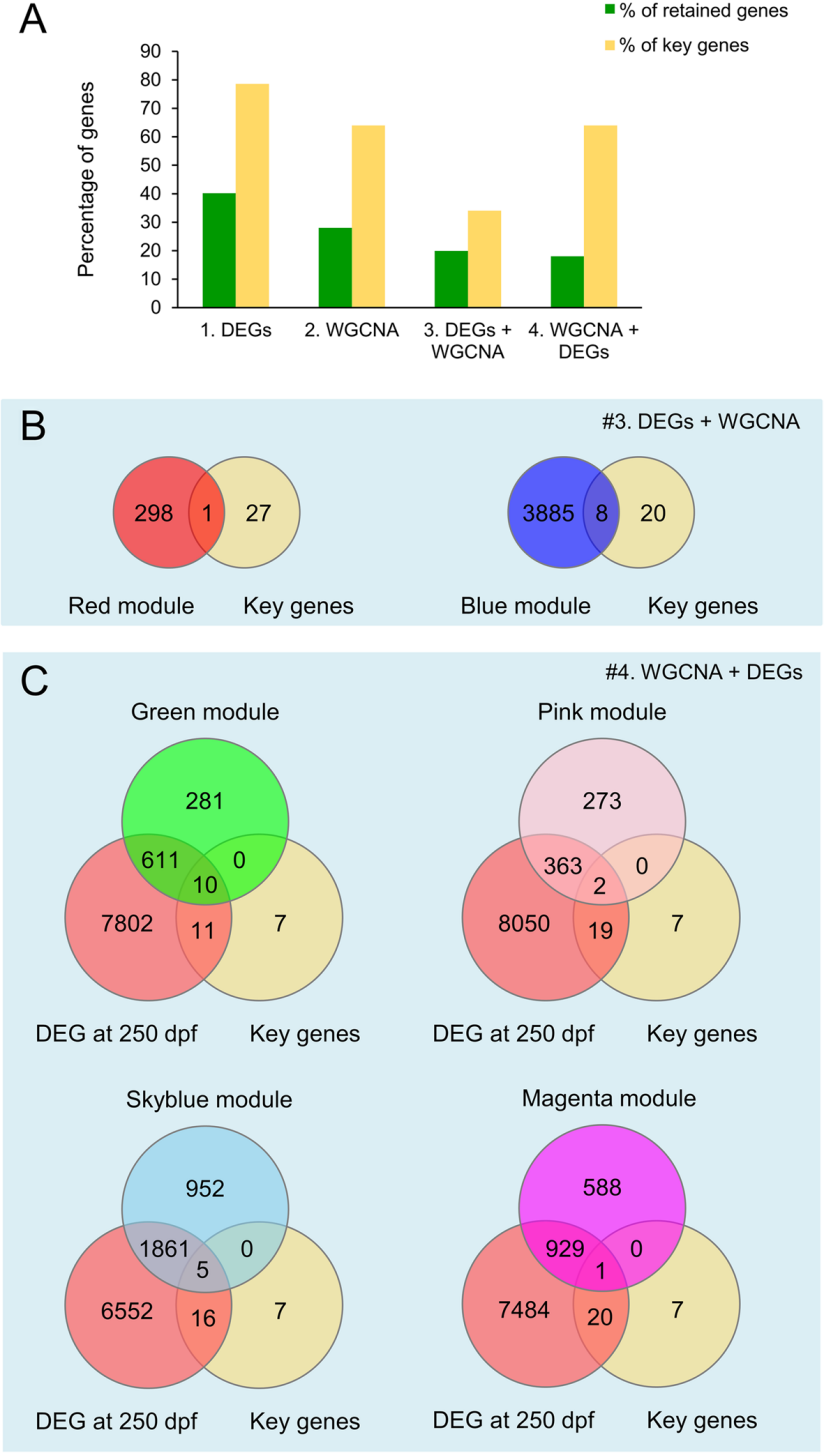
Identification of key genes involved in sex differentiation in the sea bass when using the different methods to analyze transcriptomic data. **A** Percentage of genes and key genes retained according to the methods mentioned in this study. **B** Venn diagram of the key genes found in each module using method #3 and **C** in method #4

We found that the proportion of retained genes and, within those, of key genes for gonadal sex differentiation among the four methods tested were similar in the sea bass and mouse (compare Fig. 5A and Supplementary Figure 9 A). When using method #1, filtering the transcriptome by DEGs, a large proportion of the transcriptome was kept. Up to 40.2% in sea bass (Fig. 5A) and 57.6% in mouse (Supplementary Figure 9 A) of the transcriptomes were differentially expressed. Among them, 78.6% of the key genes previously known to be involved in sexual development were found in the sea bass, and 78% of them were identified in the mouse when using method #1.

When using method #2 for the same datasets, we obtained smaller lists of genes being related to sex. In sea bass, we found that 28% of the transcriptome was involved, of which 64% of the key genes were identified. Similarly, in mouse, 22% of the transcriptome was co-expressed and associated with sex and 76% of the key control genes were detected. The third method, the application of DEG filter previous to network construction, leads to slightly smaller gene lists: 19.9% in the sea bass, and 20% of the mouse transcriptome. However, the proportion of key genes detected by this method was much smaller in the sea bass (34%, 1 gene in the red module and 8 genes in the blue module, Fig. 5B) and in the mouse (50%, 25 genes, Supplementary Figure 9 B). Finally, when using the proposed method #4, to perform WGCNA first and to apply the DEG filter afterwards, it provided us with the smaller proportions of potential novel genes related to sex trait (18% in sea bass and 15.6% of the transcriptome in the mouse) without harming the capability to detect the key genes previously known to be involved in sex development. In sea bass, 64% of the key genes were identified (Fig. 5A, C), and in mouse, 58% of the key genes were captured (Supplementary Figure 9 A and  C).

**Fig. 6:**
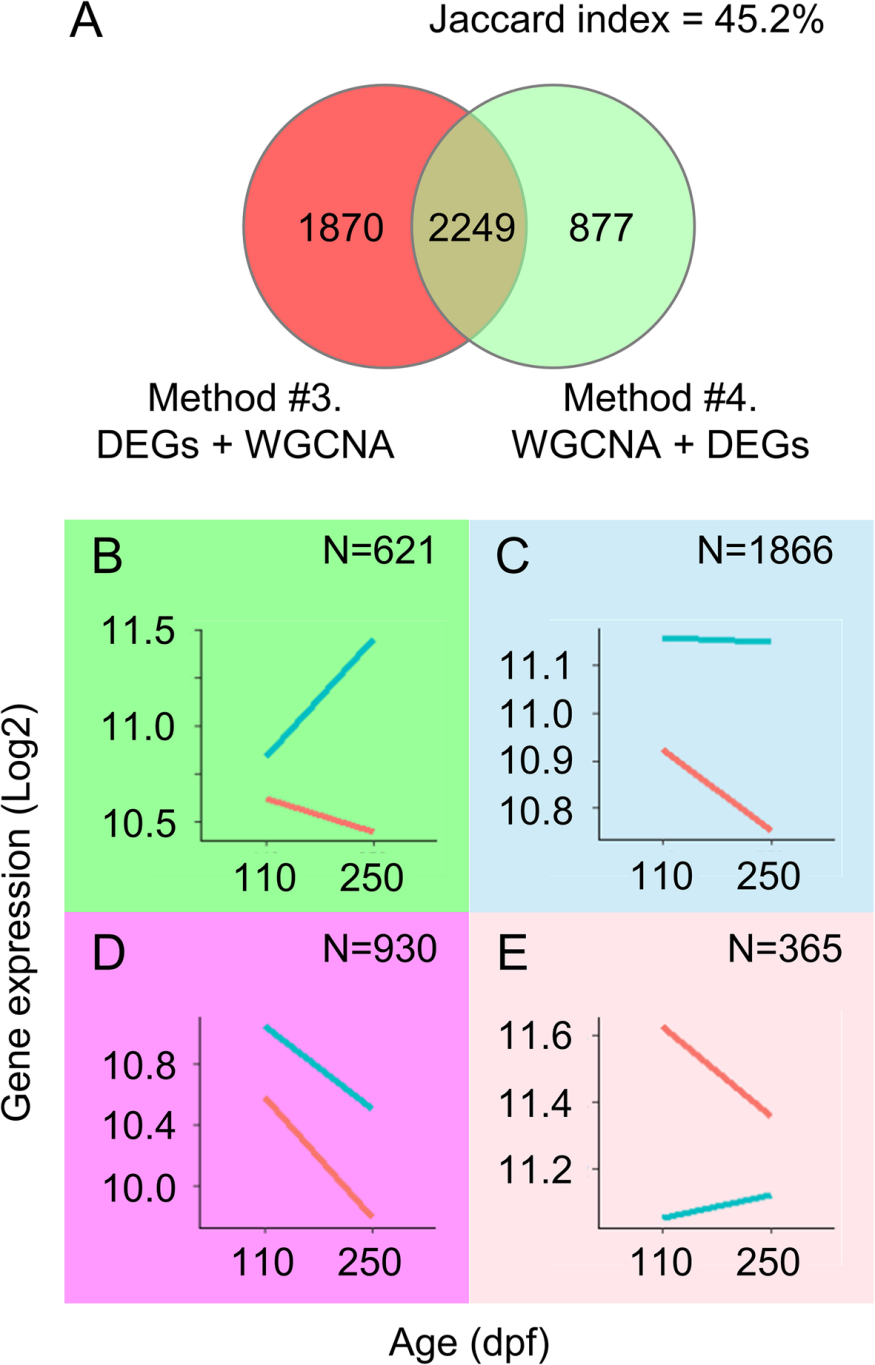
**A** Comparison of the gene lists obtained from the sea bass dataset when using method #3 and #4. The Jaccard index indicates low similarity between gene lists. Gene expression profile of the co-expressed DEGs from the four modules associated with sex and filtered by method #4. The background color of the plots indicates the color of the modules they belong to: **B** green, **C** sky blue, **D** magenta, and **E** pink module. **G** Gene expression of *sox9a* in males (blue) and females (red) at 110 and 250 dpf

It is important to note that the lists of genes produced by methods #3 and #4 differ. Method #3 yielded a total of 4192 co-expressed genes in sea bass, 1870 of which were uniquely detected using this method. On the other hand, method #4 produced a total of 3782 genes, of which only 877 were unique to that method (Fig. 6A). In the mouse dataset, method #3 detected a total of 2835, of which 1023 were unique to that method, while method #4 detected 2205, of which 393 genes were uniquely detected by this method (Supplementary Figure 9 D). Furthermore, the Jaccard index revealed low similarity between the gene lists obtained from methods #3 and #4 (45.02% in sea bass, and 56.13% in mouse).

### Network visualization and gene expression profiles of the selected genes

The data presented so far indicates that our proposed approach (method #4) gives more meaningful results of the biological process being studied than the currently used method #3. To further explore its advantages, we used the gene lists obtained with method #4 in sea bass for further network visualization and plotted the mean gene expression profiles of the chosen modules. Interestingly, the average gene expression profile of the genes making up the four significant modules showed clear differences in their temporal dynamics during sea bass sex differentiation between 110 and 250 dpf. Thus, co-expressed genes of the green module were upregulated in males and downregulated in females, reaching very disparate expression levels (Fig. 6B). Genes of the sky blue module did not change expression in males but were actively downregulated in females (Fig. 6C) while genes of the magenta modules were downregulated in both sexes (Fig. 6D). Finally, genes of the pink module started upregulated in females but were strongly downregulated by 250 dpf, while in the males their expression increased slightly (Fig. 6E).

**Fig. 7:**
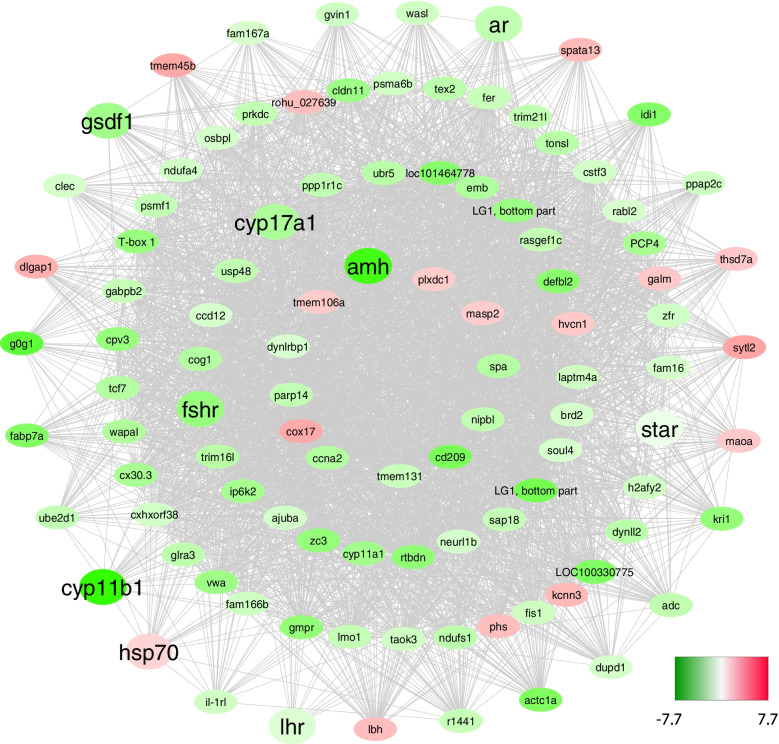
DEGs co-expression network of the green module using method #4 in the sea bass. Downregulated genes at 250 days post fertilization in females are shown in red and upregulated genes in males are indicated in green (in fold change, FC). The position of the nodes or genes indicates the degree range of each gene, which is higher to lower from inside to outside of the net. Key genes (*N* = 9) are shown in bigger nodes and written in bold

Genes making up the green module (*n* = 621) were those whose expression levels differed the most over time. For representation purposes, Fig. 7 shows only a subset of these genes (*n* = 102) having the highest number of connections (width > 0.3). From the inside to the outside, the four concentric circles show the genes with a higher degree (number of connections), including Cytochrome C Oxidase Copper Chaperone COX17 (*cox17*), Cyclin A2 (*ccna*), Cytochrome P450 Family 11 Subfamily A Member 1 (*cyp11a1*), Ajuba LIM Protein (*ajuba*), and Proteasome 20S Subunit Alpha 6 (*psma6b*). Importantly, 18 of the 28 key genes selected for their role in sea bass gonadal sex differentiation were represented in the different modules. Nine of them were in the green module with the highest degree (from higher to lower): Anti-Müllerian Hormone (*amh*), Cytochrome P450 Family 17 Subfamily A Member 1 (*cyp17a1*), Follicle Stimulating Hormone Receptor (*fshr*), Steroidogenic Acute Regulatory Protein (*star*), Androgen Receptor (*ar*), Gonadal soma derived factor (*gsdf*), Heat Shock Protein Family a Member (*hsp70*), Cytochrome P450 Family 11 Subfamily B Member 1 (*cyp11b1*), and Luteinizing Hormone/Choriogonadotropin Receptor (*lhr*).

In the magenta module, the key gene found was Hydroxysteroid 17-Beta Dehydrogenase 10 (*hsd17b10*), and some of the genes with the highest degree were Vimentin (*vim*), Protein Kinase C and Casein Kinase Substrate in Neurons 3 (*pacsin3*), and Hephaestin Like 1 (*hephl1*) (Supplementary Figure 10).

In the pink module, two of the key genes were found among the selected genes and with the highest degree of connectivity: Estrogen Receptor beta 2 (*erb2*), and Hydroxy-Delta-5-Steroid Dehydrogenase, 3 Beta- And Steroid Delta-Isomerase 1 (*hsd3b*). Similarly, other genes included were as follows: Aquaporin 10 (*aqp10*), Zinc Finger AN1-Type Containing 3 (*zfand3*), POP4 Homolog, Ribonuclease P/MRP Subunit (*pop4*), Tubulin Alpha 8 (*tuba8*), Cytochrome C Oxidase Subunit 5A (*cox5a*), and Acyl-CoA Oxidase 3, Pristanoyl (*acox3*) (Supplementary Figure 11). Lastly, with the chosen degree threshold for better network visualization, fewer genes were shown in the sky blue network, including Ubiquitin-Specific Peptidase 5 (*usp5*), Keratin 8 (*krt8*), and Schwannomin Interacting Protein 1 (*schip1*) (Supplementary Figure 12).

### Prediction of biomarkers of early gonadal differentiation in the European sea bass

**Fig. 8:**
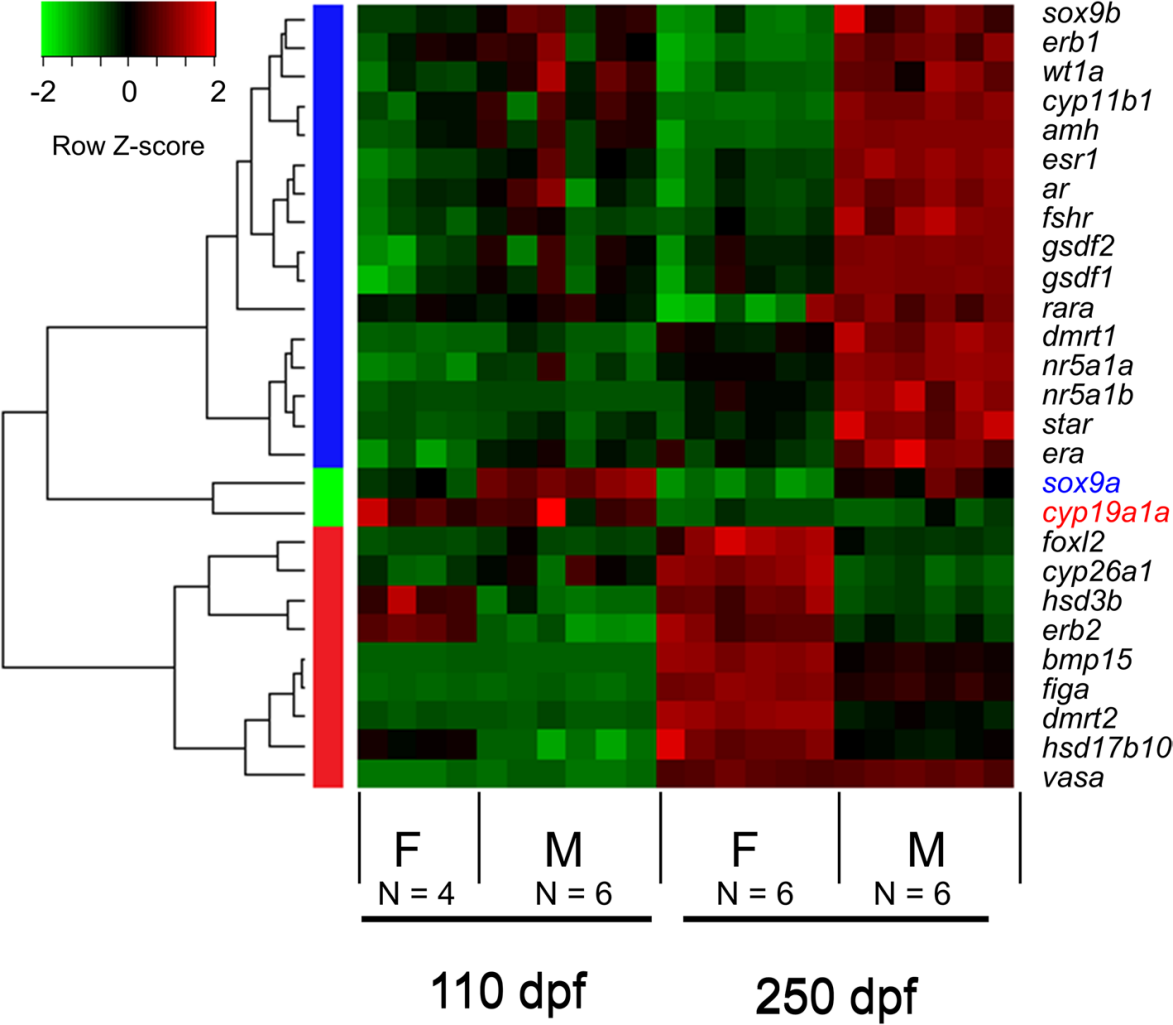
Hierarchical clustering analysis and heat map of the key genes in the sea bass. Upregulation is indicated in red and downregulation is shown in green. The left bars indicate the three main gene expression patterns found. The blue bar includes the genes upregulated in males at 250 dpf, the red bar includes the genes upregulated in females at 250 dpf while the two genes included in the green bar are those identified as early biomarkers, which show a different gene expression pattern already at 110 dpf between males and females

Since the sea bass has PSD, and thus there are no genetic markers for sex, we wanted to focus on genes involved in the process of gonadal sex differentiation to see if we could at least identify robust markers of the early stages of this process. To do so, we used the gene lists obtained from WGCNA built from the entire transcriptome and filtered the output with DEGs only at 110 dpf. We obtained a total of 2089 genes which were strongly correlated to sex. Among these genes, two of the key genes found were *hsd17b10,* in the magenta module, and the ortholog of SRY-Box Transcription Factor 9 a (*sox9a*), which was detected in the sky blue module. Furthermore, hierarchical cluster analysis of the 28 key genes during the sea bass gonadal sex differentiation showed three main gene expression patterns: (1) genes upregulated in males at 250 dpf, (2) genes upregulated in females at 250 dpf, and (3) genes upregulated in males or females already at 110 that increase their expression by 250 dpf (Fig. 8). *sox9a* was clustered together with *cyp19a1*, the already described biomarker in previous studies for ovarian differentiation. They were clustered together according to the third gene expression pattern mentioned above. Altogether, this suggested that *sox9a* expression could be used as early marker of male sex differentiation in the sea bass.

### Prediction of biomarkers of early gonadal differentiation in the mouse

In the case of the mouse, there is no need for biomarkers for sex because they have genetic sex determination (GSD) with sexual chromosomes, which allow identifying the sex as early as the zygote stage. However, we used methods #2 and #4 to test their performance in biomarker identification using a well-known model. For this objective, method #1 was not used because it generated lists of several thousand candidate genes from which it was impossible to choose a few candidates. Similarly, method #3 was discarded because of the observed bias in the results. However, when using method #2 in the brown module, for example, we found a total of eight key genes. When using method #4, the number of potential biomarkers of sex at the first stage was five genes: Wnt Family Member 4 (*wnt4*), Fibroblast Growth Factor Receptor 2 (*fgfr2*), Bone Morphogenetic Protein 2 (*bmp2*), Follistatin (*fst*), and GATA Binding Protein 6 (*gata6*). The three genes filtered out by method #4 were not DEG at the E11.0–12.0 stage between males and females but were associated with sex by WGCNA. Such genes are relevant for sex development in the mouse but not among the first to show differential expression between males and females.

Therefore, the filter by DEGs in method #4 is essential to increase the probability of finding genes that could show differential gene expression when compared to method #2. This allows the prediction of the sex on other individuals based on the gene expression measurement of a biomarker. Hence, method #4 was the approach that allowed obtaining the shorter lists of candidates meeting all the criteria from WGCNA and, on top, they were among the first to show differential expression at the stage of interest.

### Comparison of the functional enrichment analysis from two different methods in the mouse dataset

Next, we performed gene ontology (GO) enrichment analysis of the filtered gene lists produced with methods #3 and #4 in mouse (the system we used that had the most curated annotation). The results showed that GO terms directly related to biological processes associated with gonadal development were more enriched and fell under a more robust significance threshold when using method #4 than when using method #3 (Additional file 5). Secondary alcohol biosynthetic process (GO:1,902,653), cholesterol biosynthetic process (GO:0,006,695), sterol biosynthetic process (GO:0,016,126), and steroid biosynthetic process are a few examples (GO:0,006,694). Furthermore, only method #4 captured the term GO cell morphogenesis involved in differentiation (GO:0,000,904).

## Discussion

Studies based on transcriptomics data can be broadly classified into three different major methods: #1 DEGs, #2 WGCNA, and #3 filtering by DEGs (or other filters such as keeping the 20% with more gene expression variance, or the top 5000 DEGs) before WGCNA [20, 48, 54, 60]. To combine the power from WGCNA with the filter from the traditional and robust statistical analysis without introducing bias, we propose a new pipeline: (4) WGCNA of the entire transcriptome filtered by DEGs only after network construction.

**Fig. 9:**
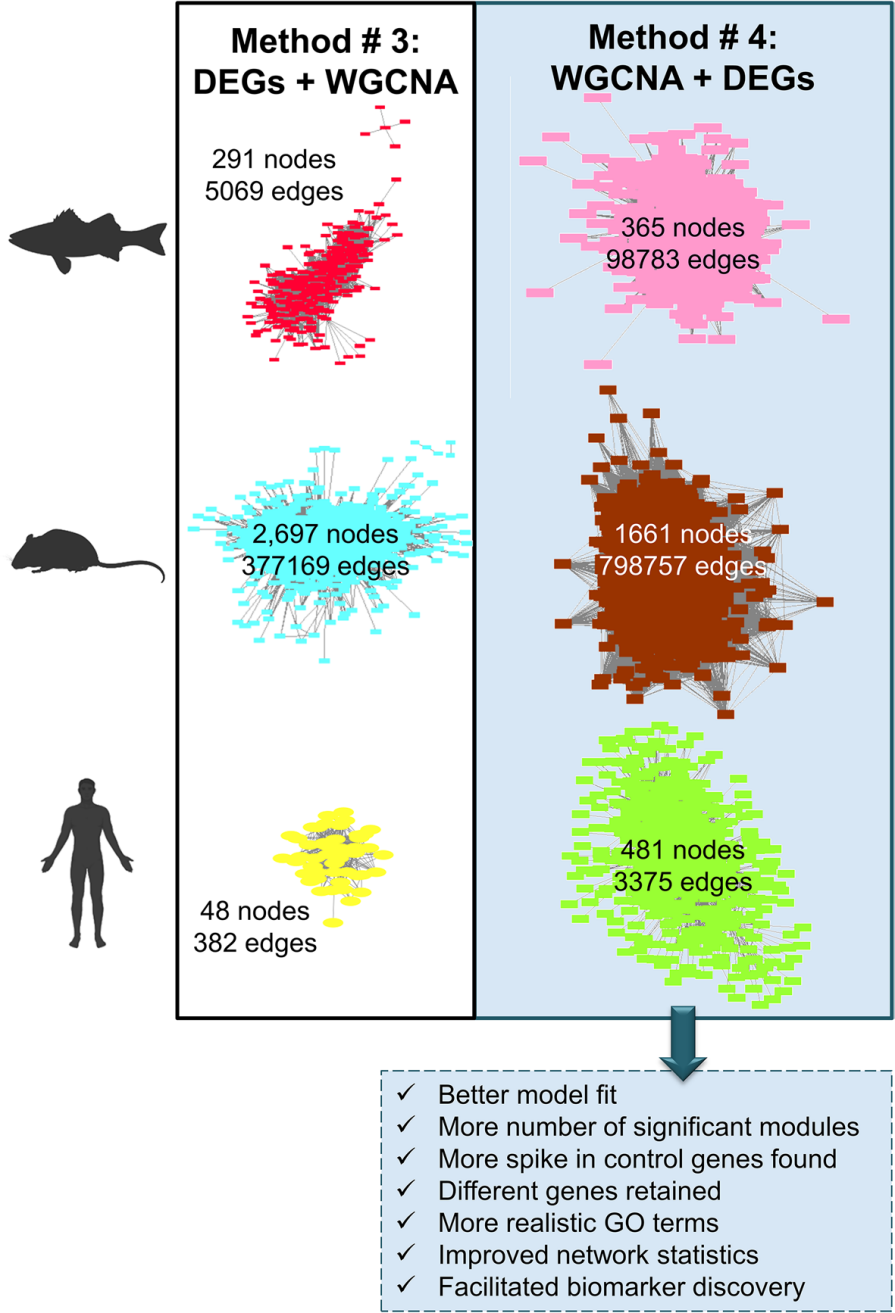
Comparison of module networks built using method #3 (DEGs + WGCNA) and method #4 (WGCNA + DEGs). The selected modules for network statistics comparison and visualization were those that showed the strongest association with sex on each method and species. Hence, we compared the modules: red vs pink in the sea bass, turquoise vs the brown in the mouse, and yellow vs the green-yellow in human. For visualization, we filtered the networks by edge weight > 0.2 threshold

In the present study, we compared the performance of filtering by DEGs before (method #3) or after (method #4) WGCNA in the analysis of one of the most transcriptomically complex organs, the gonads, during the critical period of sex differentiation. And to do so, we used three different vertebrates, a modern fish, the mouse model, and human. The fish has a very different sex-determining system than the mouse and the human that, in contrast, have a very similar sex-determining system. In all species, method #4 (WGCNA + DEGs) clearly and consistently outperformed method #3 (DEGs + WGCNA) (Fig. 9). WGCNA establishes an adjacency matrix of correlation values which is transformed using a power function. The value used to power the matrix is chosen based on parameters that ensure a scale-free topology (in order of importance): signed *R*^2^ ≥ 0.8, or the *β* value which maximizes a scale-free independence, high mean connectivity, and the slope (− *γ*) of the regression line between log_10_(*p*(*k*)) and log_10_(*k*) was around − 1. In sea bass, the best scale-free topology model fit using method #3 resulted in index *β* = 9 leading to *R*^2^ = 0.7, low mean connectivity, and the − *γ* slope of − 0.47. However, using method #4, for the same dataset, a much better scale-free topology model fit (*R*^2^ = 0.87) was obtained with a lower power index, *β* = 5. Also, higher mean connectivity and − *γ* was slightly closer to − 1. In mouse, using method #3, even a high soft threshold index of *β* = 14 lead to a weak model fit *R*^2^ = 0.55, low mean connectivity, and the slope of the regression line between log_10_(*p*(*k*)) and log_10_(*k*) of − 0.79. Using method #4 with the same dataset improved all the parameters during co-expression network construction. The *R*^2^ increased from 0.55 to 0.7, and the mean connectivity increased from hundreds to a few thousands. Furthermore, the − *γ* slope decreased from − 0.79 to − 0.88. The improvement of model fit was also observed in human using method #4 (*β* = 8, *R*^2^ = 0.89, mean connectivity > 200, and − *γ* =  − 1.38 (*R*^2^ = 0.87) when compared to method #3 (method #3: *β* = 8, *R*^2^ = 0.67, mean connectivity value < 50, and − *γ* =  − 1.02 (*R*^2^ = 0.63). The results from six analyses (using two methods in three species) under different soft threshold values showed that the network model fit is much better when using method #4, objectively.

Another important outcome of our comparison related to the network construction is the number of modules created by each method. Thus, method #3 created 8 modules while method #4 created 29 in sea bass. In the mouse, these figures were 6 and 21, and in human 8 and 36, respectively. These results consistently show that the number of modules associated with the trait of interest is considerably smaller when using method #3, clearly showing that potentially interesting genes and connections are lost already before WGCNA is performed, diminishing its power. These results were consistent with values found in the literature regardless of vertebrate species or question being answered, where method #3 produces fewer modules (5 modules [46]; 12 in [47]; and 6 in [48], than studies using WGCNA without previous filtering (17 modules in [40]; 23 in [36]; and 14 in [44]).

Several strategies exist to compare gene co-expression networks [53, 71]. We calculated topological parameters for comparison of the network module most associated with the trait of interest using methods #2, #3, and #4. All the network parameters from method #4 outperform method #3 in two or all the three species studied. One of the instances was the characteristic path length in human, which showed a shorter path length in the network built using method #3. This could be because of the much smaller number of nodes obtained with such a method, which could provide a shorter length despite being a less compacted network, as noticed elsewhere [69]. Some of the other parameters that did not clearly improve using method #4 compared to method #3 resulted from the mouse. Such networks were built with 11 samples (less than the minimum sample size recommended by the developers of WGCNA) [61]. Therefore, results should be considered with precaution. We observed in the literature that out of 20 studies in Table 1 using WGCNA or DEGs + WGCNA, some used *n* < 15 (*n* = 13 [44]; *n* = 10 [43]; *n* = 8 [48]; *n* = 14 [60], and *n* = 8 [52]), although the majority used > 15 samples to construct robust WGCNA (*n* = 160 [36]; *n* = 183 [40]; *n* = 25 [42], *n* = 430 [20], *n* = 45 [58], *n* = 36 [32], *n* = 228 [31], *n* = 48 [27]). Considering the developers’ recommendation and our results, we take the opportunity to further emphasize that at least 15 samples in total are required to perform method #2 and extend this recommendation to method #4.

Regarding the results of biological relevance, which was to identify genes related with sex and therefore with reproduction, higher proportions of genes previously known to be involved in sex differentiation were retained for further analysis when screening with method #4. Of note, not only the number of genes captured was larger in both species, but also essential key genes in a given process (here used as key controls), described in previous works, were also better represented with method #4. For example, key genes found in sea bass were as follows: *amh* [72], *gsdf* [73], *ar* [74], and *erb* [75]. Similarly, some of the key genes found in mouse were as follows: *dmrt1* [76], *amh* [77], *ar* [78], *foxl2* [79], *bmp6* [80], and *wnt4* [81]. These genes were retained with method #4 but not with method #3. Thus, our approach results in a more meaningful representation of the biological question under consideration.

Another important insight of this study is that we clearly show that methods #3 and #4 generate different gene lists. As a result, this has an impact on the downstream functional analysis and can affect the resulting GO biological processes, as well as the interpretation of the results (Additional file 5). First, the overrepresented GO biological processes from the gonadal transcriptome of the mouse at 16.5 dpc slightly varied depending on the filtering strategy and the order in using such filters leading to different results. First, we found that GO terms from the category of biological processes related to gonad development functions were more enriched and had a higher significance threshold when using method #4. Second, only method 4 yielded some terms related to the gonadal sex differentiation process. Altogether, these results provide evidence that more accurate gene lists related to the target phenotypic trait were achieved using method #4 (WGCNA + DEGs) than when using the DEG filter or gene lists from pre-filtered WGCNA as in method #3.

We are aware that the results from gene ontology enrichment analysis should not be considered proof of biological validity in the analysis of high-throughput data considering the multiple sources of bias existing in functional enrichment analysis tools [82]. However, to date, this is one of the main methods used to summarize information from high-throughput experiments and here we showed how the previous filtering of DEGs can affect the results of WGCNA.

In search of early sex differentiation markers in the sea bass, several genes have been identified so far in the literature. Blázquez et al. [83] defined *cyp19a1a* as a suitable molecular marker of ovarian differentiation, which showed the first significant gene expression difference between sexes at 120 dph. Ribas et al. [35] identified the *hsd17b10* gene as an early marker for ovarian differentiation at 110 dpf. At this stage, *cyp19a1a* expression levels were higher in females but not significantly different from males. In the present study, among the key genes involved in early gonadal development, three genes stood out from the combined filter of WGCNA and the subsequent DEG filter at 110 dpf. Among these three genes, *hsd17b10* was found, hence, supporting the results from Ribas et al. [35]. The other gene identified as marker of testis early differentiation was s*ox9a*. Noteworthy, this gene could not be identified in previous studies using method #1 [35].

*sox9* is a multifunctional transcription factor found in different tissues and plays crucial roles in vertebrate development, including cell proliferation and differentiation [84]. This gene was defined as a “hub” gene of testis differentiation after sex determination in vertebrates. As reviewed elsewhere [84], *sox9* has a conserved role in male gonadal development and a highly conserved protein sequence. In fishes, two orthologs exist due to gene duplication: *sox9a* and *sox9b* [85]. Although the two orthologs are retained, they are related to different functions and tissues depending on the fish species. For example, *sox9a*, is expressed in the ovary of the medaka (*Oryzias latipes*) and the platy fish (*Xiphpphorus maculatus*), while in the zebrafish (*Danio rerio*), *sox9a* is expressed only in the testis [85], like the current results found in the sea bass. In this species, *sox9a* was recently found to be involved in the epigenetic regulation of the temperature induced sex ratio during sex differentiation [86].

Based on the parameters obtained in the model fit during network construction, network statistics, and the biological relevance of the results obtained, we highlight the importance of using method #4 followed by functional enrichment analysis rather than method #3. Even though the computation of method #3 (DEG + WGCNA) is faster (a matter of seconds to a few minutes for the datasets used in this study) and requires less computational power, it does not compensate for the bias introduced to the results. Additionally, we recommend using method #4 instead of method #2 because, although the same network is built and the same groups of genes are formed, method #4 allows to further filter the large gene lists produced by WGCNA without removing the most important (based on gene expression) genes. WGCNA is a powerful method still being used [20, 42–44], with a large proportion of the studies using specifically the inappropiate method #3 [54–60]. More recent alternative methods to WGCNA to identify modules of differentially co-expressed genes have been developed: THD-Module Extractor [22], DiffCoEx [23], and MODA [24]. And, although in the comparison of these methods with WGCNA, the THD-Module Extractor method was claimed more effective in finding modules with higher functional relevance and biological significance than WGCNA, this result could be due to the previous filter of the dataset. Precisely, as stated by the authors, the use of DEGs before WGCNA lead them to the soft threshold of power fit to fail [53]. Therefore, a comparison of THD-Module Extractor method with the method #4 (WGCNA + DEGs) described in this study remains to be done.

## Conclusions

In conclusion, WGCNA is a robust and systematic approach commonly used in transcriptomics. With time, several strategies have appeared and became quite common, i.e., method #3, to use the parameters and filters provided by this tool but somehow running against its original philosophy. We evaluated the model fit and the biological relevance of the results obtained using method #3 (DEGs + WGCNA) and our proposed method #4 (WGCNA + DEGs) with the gonadal transcriptome of three different vertebrates, sea bass, mouse, and human, obtained from two high-throughput technologies: microarray and RNA-seq. The results showed that method #4 is more efficient in filtering for smaller gene lists that contain the genes most related to the trait of interest. Thus, we propose to analyze transcriptomic data using WGCNA to build a co-expression network from the entire dataset and, only subsequently, filter by DEGs. Such a strategy combines the powerful method of producing a network with the filter from the traditional and robust statistical analysis without introducing bias. Additionally, we produced new lists and network visualization of genes related to early sex differentiation in the sea bass with the corresponding functional enrichment analysis and identifying a novel biomarker for testis early differentiation (*sox9a*). Last but not least, the filter by DEGs in method #4 increases the probability of robust biomarker discovery when compared to method #2. We propose that method #4 should be the method to use in future transcriptomic studies regardless of biological system, species, or question being considered.

## Methods

After WGCNA development, numerous studies started to use this method in combination with the previous filtering of the dataset (method #3). Combining WGCNA with other filtering methods like DEGs can be a strategy to further filter for the target genes associated with a trait of interest. However, whether this filtering step before WGCNA affects the results and to what extent has never been shown yet. In this study, we aim to compare method #3 with the here proposed new method #4: applying the filtering step after construction of the co-expression network. To determine which one is more efficient, we used both methods to study the gonadal transcriptome of three vertebrates: sea bass, mouse, and human. Hence, a total of six analyses were performed. Then, we compared the model fit, connectivity, and other network statistics to compare both methods for each of the three species. Additionally, we used existing information on key genes for the sexual development and reproduction of sea bass and mouse to measure how many key genes were found with each method. Finally, the enriched genes by GO term enrichment analysis obtained from the gene lists produced by methods #3 and #4 were compared in the mouse. The methods are implemented in R software and all source code has been made publicly available on GitHub as part of the WGCNA_DEGs project at: https://github.com/Nsbaizan/WGCNA_DEGs.

### Literature review

Before any comparison of methods #3 and #4 was performed, we wanted to know if the use of method #3 was exceptional or if we could find several studies where this method was used. We used Scopus and Web of Science databases to find published studies using each method. To search a few examples of method #1, we used the following keywords in the topic field: “((DEG) OR (“differentially expressed genes”)) AND ((Microarray) OR (RNA sequencing))”. To find studies using methods #2 or #3, we used the following keywords in the topic field: “((WGCNA) OR (“weighted gene co-expression network analysis”)) AND ((Microarray) OR (RNA sequencing))”. After reading detailed information on the methods section of each paper, we could classify between methods depending on whether WGCNA was used without (method #2) or after filtering by DEG (method #3). We found that the use of the WGCNA is widespread in transcriptomic data studies and that method #3 is not exceptional but, rather, it is of common use even in recent publications.

### Species and processes studied: the sea bass

In sea bass, sex depends on the combination of several pro-male and pro-female autosomal genes plus environmental (temperature) influences [64]. Sex determination is thought to occur between 60 and 100 dpf [87], around 120 dpf the first molecular signs of gonadal sex differentiation appear in the form of differences in the expression of gonadal aromatase, *cyp19a1a* [88], and the first histological differences appear around 150 dpf when fish are around 8 cm long. Sex differentiation proceeds earlier in females than males, and the process is completed by 250 dpf in females and around 350 dpf in males when females are 12.7 ± 5.7 cm (mean ± SD), and 11.2 ± 0.6 cm of SD in males, and 16 ± 1.3 in females and 14.8 ± 1.1 cm of SD in males, respectively [35]. Gene expression during gonadal sex differentiation in the sea bass has been extensively studied, first by targeted approaches [89–92] and more recently by using a homologous and validated microarray containing 43,803 probes. In the latter case, fish before (110 dpf), during (250 dpf), and after (350 dpf) gonadal sex differentiation were analyzed [35], and DEGs and enriched signaling pathways were identified. Additionally, *hsd17β10* was identified as a marker of early ovarian differentiation.

### Species and processes studied: the mouse

Sex differentiation in the mouse is well characterized [93] and thus less information will be provided. Briefly, key genes during mouse gonadal sex differentiation include sex-determining region Y (*sry*), *amh*, and *sox9*, while pro-female genes include *wnt4*, R-spondin 1 (*rspo1*), a member of the R-spondin family, and β catenin (*ctnnb1*) [93]. In mouse, sex differentiation starts between embryonic day (E) 11.0–E12.0 and ends at E16.5 [94, 95].

### Species and processes studied: the human

Sex differentiation in the human has been studied extensively despite the limited availability of normal fetal human gonads [66]. In brief, the gonadal primordium arises from the coelomic epithelium around the 4th PCW and differentiation reaches the end towards the 17th PCW. Testis differentiation is activated by the expression of the Y-linked transcription factor *sry* during the 6th PCW in supporting cells leading to the expression of *sox9* and *amh* genes. In the absence of *sry*, *rspo1*/*Wnt4*/β catenin pathway, *foxl2* activates the transcriptional cascade required for ovarian differentiation.

### Datasets

Transcriptomic data during sea bass sex differentiation was previously obtained using a homologous microarray [35] and can be downloaded from the Gene Expression Omnibus (GEO) database with the accession number GSE115841 [96]. For the aim of the present study, we selected data available from fish at two key developmental stages: four females and seven males at the beginning of sex differentiation at 110 dpf with a length of 5.2 ± 0.5 cm (mean ± SD). At this time, gonads are still morphologically undifferentiated but fish can be sexed measuring *cyp19a1a* expression levels [88]. We also used six females (12.7 ± 5.7 cm) and six males (11.2 ± 0.6 cm) in the middle of sex differentiation period at 250 dpf. Thus, we used 23 fish in total for analysis. The original downloaded file consists of 43,801 probe copies representing 20,978 transcripts with normalized expression values, corrected for batch effect. Microarray intensity values were directly used for the determination of differentially expressed genes and/or network construction.

Transcriptomic data during mouse sex differentiation was obtained using RNA-seq [96] and can be downloaded from the GEO database under the accession number GSE117590 [97]. For the present study, we selected transcriptomic data from twelve samples, three males and three females at two embryonic stages: 12.5 dpc, corresponding to the beginning of gonadal sex differentiation, and 16.5 dpc, at the end of sex differentiation. After trimming the raw reads, the alignment to reference genome (v. GRCm39 GCA_000001635.9) (2.1.0) [98] was performed to obtain a dataset with 55,416 genomic features using featureCounts (v2.0.0) [99]. Counts were processed using edgeR package (v3.34.0) [100]—limma workflow which includes counts pre-processing and exploratory data analysis before obtaining lists of DEGs as described in [101].

Transcriptomic data during human sex differentiation was obtained using RNA-seq [66] and can be found at NCBI GEO under accession number GSE116278 [102]. We selected transcriptomic data from a total of 32 samples which were grouped into three stages as follows: four males and four females at 6 PCW, corresponding to the beginning of gonadal sex differentiation (first stage); 8 males and 8 females at 7 PCW (second stage); as well as two males and two females at 13–14 PCW, and two males and two females at 17 PCW, corresponding to the end of sex differentiation (third stage). The total of 35,194 finely annotated transcripts in [66] were used to test methods #3 and #4.

### Transcriptomic data analysis overview

The gonadal transcriptome of the sea bass, mouse, and human during sex differentiation were analyzed with two different methods: #3 (DEGs + WGCNA) and #4 (WGCNA + DEGs). In method #3, we first created a list of the DEGs between males and females at 250 dpf in sea bass and at 16.5 dpc in mouse and then we used this list for network construction. For the human, we used exactly the same list of differentially expressed transcripts between males and females at 6 PCW previously published [66]. In method #4, we first carried out WGCNA and then used the same list of DEGs as before to filter the dataset just after network generation but prior to network visualization. Therefore, we carried out a total of six analyses (two methods × three species). In the following sections, we describe in detail how we determined the DEGs and how we carried out WGCNA in each case.

### Weighted gene co-expression network analysis

We implemented the WGCNA using the R package (v.1.51) according to the authors’ recommendations [17, 103] in R statistical software [104, 105]. We first checked for the presence of outliers using the hierarchical clustering of samples with Euclidean distance [68]. In the sea bass dataset, one male at 110 dpf grouped with the male samples at 250 dpf at a height > 15, distant from the rest of samples and thus removed from further analysis. The rest of the samples were used to generate the Pearson correlation matrices. Among the mouse samples, one was identified as an outlier using the hierarchical clustering of samples. One of the females at 16.5 dpc was at a height > 30 far from the rest of samples and hence, removed from further analysis. Thus, in total we analyzed 22, 11, and 32 samples from sea bass mouse, and human, respectively.

To build unsigned weighted networks, the adjacency matrix was calculated as *a*_mn_ =|cor_mn_|*β*, where *a*_mn_ is the adjacency between gene m and gene n, cor_mn_ is the Pearson correlation, and *β* is the soft-power threshold. Unsigned networks allow the connection of genes that are both positively and negatively correlated; the absolute value of the Pearson correlation is used as a co-expression similarity measure [106, 107]. In contrast, a signed network would not include the connections between strong negatively correlated genes [106, 107]. Sexual development in vertebrates is known to be orchestrated by mutually antagonizing male and female pathways [63] in which not only upregulation of a set of genes tilts the balance towards the development of one sex or the other but, importantly, the concomitant mechanism of active downregulation of numerous genes in the opposite sex is also required [95, 108]. Therefore, using the unsigned network allowed drawing the connections between genes or nodes that were either positively or negatively correlated, since no difference was made between gene inhibition and activation patterns.

To fit the scale-free topology model, we tested several soft threshold powers (ranging from 1 to 20) to which co-expression similarity is raised. To quantify how well a network met the scale-free topology criterion, the model fit was measured as the signed linear regression model fitting index *R*^2^. After selection of the value leading to the best fit to the scale-free topology model (signed *R*^2^ closest to 1), additional considerations described by the developers were taken into account: high mean connectivity (*k*), and the slope of the regression line between log10 (*p*(*k*)) and log10 (*k*), closest to − 1 [17, 103]. Subsequently, the adjacency matrix was transformed into a topological overlap matrix, and gene modules were detected by hierarchical average linkage clustering analysis for the gene dendrogram, setting the parameters as default (minimal gene module size = 30, and the threshold to group similar modules was set to 0.25).

### Selection of modules associated with sex

After the modules were defined, the module eigengene (ME) distances were calculated to elucidate potential relationships of modules with two phenotypic traits: sex and age of the samples. For simplicity, we focused on the results related to sex development in the present study, although the study of other traits could be considered following the same workflow. Hence, we chose the significant modules that met the following thresholds regarding sex trait: absolute *R*^2^ > 0.5 and *P* < 0.001 in the sea bass and human datasets or *P* < 0.01 for the mouse transcriptome. To assess the correlation strength, we calculated the module significance (MS), the average absolute GS of all the genes involved in the module. The key modules kept for further analysis were those with the highest MS score among all modules produced.

For the genes within the selected modules, we calculated intra-modular membership (*k*_IM_) for each gene to determine how well-assigned is a gene within a module and its relationship with the trait of interest as described by Langfelder and Horvath [17]. Additionally, we calculated GS for the sex trait of each gene within the modules. The gene significance is defined as − log of the *p*-value of association of the gene with the trait, in our case, sex.

To identify the modules with most interesting genes associated to the trait of interest, we selected the modules with a significant correlation (|cor|≥ 0.5, *P* < 0.001 in sea bass and human; |cor|≥ 0.5, *P* < 0.01 in mouse). To identify the genes most interesting within those modules, we filtered by *k*_IM_ and GS, where the higher the absolute value of GS of a gene the more biologically significant it is for the trait of interest. Thus, we kept for further analysis all the genes within the selected modules with an absolute gene significance higher than 0.2 and an absolute intra-modular membership higher than 0.8 [17].

### Analysis of differentially expressed genes

The DEGs were determined by fitting a linear model, using the same empirical Bayesian statistics in both datasets. For the sea bass data, we used the Quantile method in the Linear Models for Microarray Analysis (Limma) (v. 3.44.3) R package [109]. We compared sea bass female vs male gonads at 110 and 250 dpf. The same statistical test was applied to the mouse transcriptome dataset using edgeR (v.3.30.3) [100] and GLimma package (v.2.2.0) [110] in R software (v. 3.4.1) [104, 105]. In this case, the comparison of female vs male at 16.5 dpc was used. For all the comparisons, genes with a false discovery rate (FDR) based on the Benjamini–Hochberg method were defined as differentially expressed (adjusted *P* < 0.05). We pre-processed the two transcriptomes using different packages because the dataset from the sea bass was obtained from a microarray experiment (normalized intensity values matrix), and the mouse data were obtained from an RNA sequencing experiment (normalized expression counts matrix). While data pre-processing steps were necessarily different because of the technologies used to obtain the data, the mathematics behind DEG determination were essentially the same.

### Key genes with reproduction-related functions

To determine which methodology would unveil more genes relevant for the target process, we generated lists of genes previously known to be involved with gonadal sex differentiation for each of the two species studied. For the sea bass, we selected a total of 28 genes from the literature related to the sea bass or other well-studied fish species like zebrafish [35, 93, 111] (Additional file 6). In addition, we produced a heat map with gene hierarchical analysis of the selected genes in sea bass using gplots (v3.1.1) [112] and ggplot2 (v3.3.5) [113] packages. For the mouse, where gonadal sex differentiation has been thoroughly studied, we selected 50 genes compiled from recent and comprehensive reviews [93, 96, 114] (Additional file 7).

Up to this step, all analyses were performed in sea bass and mouse species to test whether the comparison between methods #3 and #4 was reproducible with transcriptomes of different species and obtained with different technologies (microarray and RNA-seq). From this point onwards, only the results from the sea bass were further explored by network visualization and identification of early gene expression markers of sex differentiation.

### Network visualization and statistics

In order to compare the three methods involving network construction, we selected the most associated module with sex (positively or negatively) to calculate network parameters. The modules were as follows: red (DEGs + WGCNA) vs pink (WGCNA + DEGs) in the sea bass, turquoise (DEGs + WGCNA) vs brown (WGCNA + DEGs) in the mouse, and yellow (DEGS + WGCNA) vs yellow-green (WGCNA + DEGs) in the human. We exported network results to Cytoscape software (version 3.5.1) [115] using the function *exportNetworkToCytoscape* from WGCNA package by including the adjacency matrix of each module as input and defining the adjacency threshold to 0.2. Cytoscape was then used to visualize and to analyze the networks using *NetworkAnalyzer* [69].

### Identification of biomarkers of early gonadal sex differentiation

To find genes involved in the early stages of gonadal sex differentiation in the sea bass, we used the gene lists of the four modules associated with sex, filtered by gene significance and intra-modular membership as described above, i.e., absolute gene significance higher than 0.2 and an absolute intra-modular membership higher than 0.8. Additionally, for those genes that met the criteria, we checked which ones were also DEGs between males and females at 110 dpf. Among the selected genes, we checked for the presence of genes previously known to be involved in gonadal sex differentiation in this species.

### Functional enrichment analysis of the mouse dataset

We chose to investigate potential differences in GO term analysis caused by different methods using the mouse, a model species with a much more curated and up to date annotation. The co-regulated DEGs associated with sex at 16.5 dpc were enriched for Gene Ontology analysis using the GO Enrichment Analysis bioinformatic PANTHER tool [116], with the list of genes captured by the RNA-seq experiment (*n* = 14,088) serving as the background reference list.

## Supplementary Information

**Additional file 1.** Table with DEGs in the differentiating gonads of the European sea bass between males and females.

**Additional file 2.** Table with DEGs in the differentiating gonads of the mouse between males and females.

**Additional file 3.** Table of co-expressed DEGs in the differentiating gonads of the European sea bass between males and females.

**Additional file 4.** Table of co-expressed DEGs in the differentiating gonads of the mouse between males and females.

**Additional file 5.** Significant GO terms enriched from the co-expressed DEGs in the differentiating gonads of the mouse between males and females.

**Additional file 6.** Key genes involved in the gonadal development of the European sea bass and other teleosts. Data compiled from [93, 111].

**Additional file 7.** Key genes involved in the gonadal development of the mouse. Data compiled from [93, 114, 117].

**Additional file 8: Supplementary figure 1.** Sample dendrogram and trait information on sex and age using hierarchical clustering analysis. **Supplementary figure 2.** Flow diagram and number of genes of methods #3 and #4. **Supplementary figure 3.** Determination of soft-threshold power in the WGCNA using the mouse gonadal transcriptome. **Supplementary figure 4.** Determination of soft-threshold power in the WGCNA using the human gonadal transcriptome. **Supplementary figure 5.** Identification of gene modules associated with sex in the mouse. **Supplementary figure 6.** Identification of gene modules associated with sex in the human. **Supplementary figure 7.** Filtering of genes based on network properties in the mouse. **Supplementary figure 8.** Filtering of genes based on network properties in the human. **Supplementary figure 9.** Identification of key genes involved in sex differentiation of mouse. **Supplementary figure 10.** DEGs co-expression network of the magenta module using method #4 in sea bass data. **Supplementary figure 11.** DEGs co-expression network of the pink module using method #4 in sea bass data. **Supplementary figure 12.** DEGs co-expression network of the sky blue module using method #4 in the sea bass data.

## Data Availability

All data generated or analyzed during this study are included in this published article, its supplementary information files and publicly available repositories. The datasets analyzed are available at the GEO database with the accession numbers GSE115841 (https://www.ncbi.nlm.nih.gov/geo/query/acc.cgi?acc=GSE115841), GSE117590 (https://www.ncbi.nlm.nih.gov/geo/query/acc.cgi), and GSE116278 (https://www.ncbi.nlm.nih.gov/geo/query/acc.cgi?acc=GSE116278).

## Abbreviations

*acox3*: Acyl-CoA Oxidase 3, Pristanoyl
*ajuba*: Ajuba LIM Protein
*amh*: Anti-Müllerian Hormone
*aqp10*: Aquaporin 10
*ar*: Androgen Receptor
*bmp2*: Bone Morphogenetic Protein 2
*ccna*: Cyclin A2
cDNA: Complementary DNA
cor: Correlation
*cox17*: Cytochrome C Oxidase Copper Chaperone COX17
*cox5a*: Cytochrome C Oxidase Subunit 5A
*ctnnb1*: β Catenin
*cyp11a1*: Cytochrome P450 Family 11 Subfamily A Member 1
*cyp11b1*: Cytochrome P450 Family 11 Subfamily B Member 1
*cyp17a1*: Cytochrome P450 Family 17 Subfamily A Member 1
DEGs: Differentially expressed genes
dpc: Days post coitum
dpf: Days post fertilization
DSDs: Disorders of sexual development
E: Embryonic day
*erb2*: Estrogen Receptor beta 2
ESTs: Expressed sequence tags
FDR: False discovery rate
*fgfr2*: Fibroblast Growth Factor Receptor 2
*fshr*: Follicle-Stimulating Hormone Receptor
*fst*: Follistatin
*gata6*: GATA Binding Protein 6
GEO: Gene Expression Omnibus
GO: Gene ontology
GS: Gene significance
GSD: Genetic sex determination
*gsdf*: Gonadal soma derived factor
*heph1l*: Hephaestin Like 1
*hsd17b10*: Hydroxysteroid 17-Beta Dehydrogenase 10
*hsd3b*: Hydroxy-Delta-5-Steroid Dehydrogenase, 3 Beta- And Steroid Delta-Isomerase 1
*hsp70*: Heat Shock Protein Family a Member
k: Connectivity
k_IM_: Intra-modular membership
*krt8*: Keratin 8
*lhr*: Luteinizing Hormone/Choriogonadotropin Receptor
Limma: Linear Models for Microarray Analysis
ME: Module eigengene
MODA: Module differential analysis for weighted gene co-expression network
mRNA: Messenger RNA
MS: Module significance
*pacsin3*: Protein Kinase C and Casein Kinase Substrate in Neurons 3
PCW: Postconceptional week
*pop4*: POP4 Homolog, Ribonuclease P/MRP Subunit
PSD: Polygenic sex determining
*psma6b*: Proteasome 20S Subunit Alpha 6
RNA-seq: RNA sequencing
*rspo1*: R-spondin 1
*schip1*: Schwannomin Interacting Protein 1
SD: Standard deviation
*sox9a*: SRY-Box Transcription Factor 9 a
*sry*: Sex-determining region Y
*star*: Steroidogenic Acute Regulatory Protein
*tuba8*: Tubulin Alpha 8
*usp5*: Ubiquitin-Specific Peptidase 5
*vim*: Vimentin
WGCNA: Weighted gene co-expression network analysis
*wnt4*: Wnt Family Member 4
*zfand3*: Zinc Finger AN1-Type Containing 3
β: Soft threshold
