# Supplementary material for: Improved biomarker discovery through a plot twist in transcriptomic data analysis
Source: BMC Biol. 2022 Sep 24;20:208. doi: 10.1186/s12915-022-01398-w (PMC9509653; doi:10.1186/s12915-022-01398-w)
Supplement: Supplementary file 8 — Additional file 8: Supplementary figure 1. Sample dendrogram and trait information on sex and age using hierarchical clustering analysis. Supplementary figure 2. Flow diagram and number of genes of methods #3 and #4. Supplementary figure 3. Determination of soft-threshold power in the WGCNA using the mouse gonadal transcriptome. Supplementary figure 4. Determination of soft-threshold power in the WGCNA using the human gonadal transcriptome. Supplementary figure 5. Identification of gene modules associated with sex in the mouse. Supplementary figure 6. Identification of gene modules associated with sex in the human. Supplementary figure 7. Filtering of genes based on network properties in the mouse. Supplementary figure 8. Filtering of genes based on network properties in the human. Supplementary figure 9. Identification of key genes involved in sex differentiation of mouse. Supplementary figure 10. DEGs co-expression network of the magenta module using method #4 in sea bass data. Supplementary figure 11. DEGs co-expression network of the pink module using method #4 in sea bass data. Supplementary figure 12. DEGs co-expression network of the sky blue module using method #4 in the sea bass data. [file 12915_2022_1398_MOESM8_ESM.docx]

**Supplementary Information**

**Increased biomarker discovery by a plot twist in transcriptomic data analysis**

Núria Sánchez-Baizán, Laia Ribas, Francesc Piferrer*

Institut de Ciències del Mar (ICM), Spanish National Research Council (CSIC), Barcelona, Spain.

Authors e-mail addresses: nsbaizan@icm.csic.es, lribas@icm.csic.es, piferrer@icm.csic.es

*Correspondence: Dr. Francesc Piferrer, Institut de Ciències del Mar (ICM), Spanish National Research Council (CSIC), Passeig Marítim, 37-45, 08003 Barcelona, Spain. Tel. +34-932309567.

**E-mail:** [piferrer@icm.csic.es](mailto:piferrer@icm.csic.es)

**Supplementary figures**

**
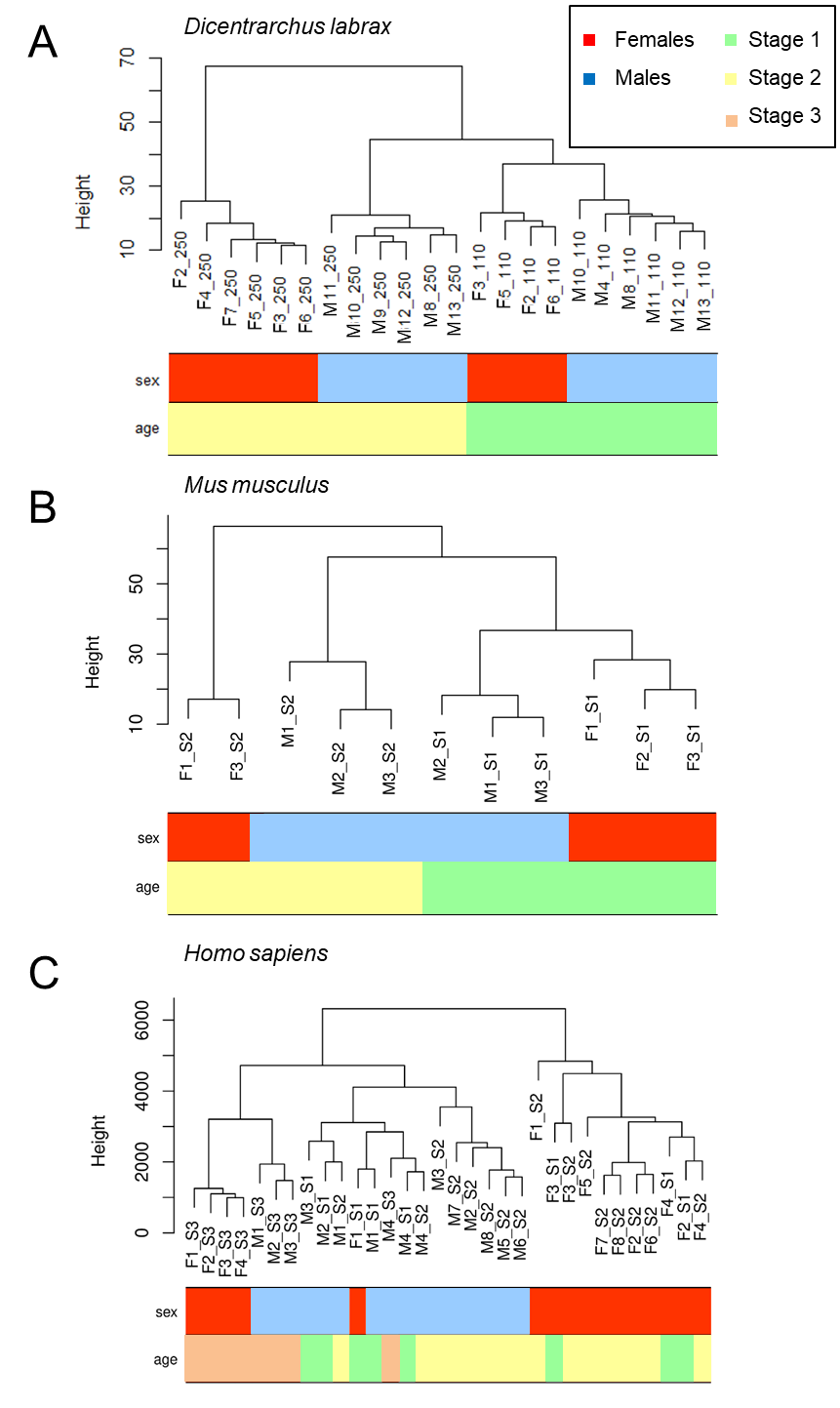
**

**Supplementary figure 1.** Sample dendrogram and trait information on sex and age using hierarchical clustering analysis. **A.** Sample dendrogram and traits of the 22 samples kept for further analysis after removal one outlier in the sea bass dataset. **B.** Sample dendrogram and traits of the 11 samples kept for further analysis after removal one outlier in the mouse dataset. **C.** Sample dendrogram and traits of the 32 samples kept for analysis from the human dataset.

**
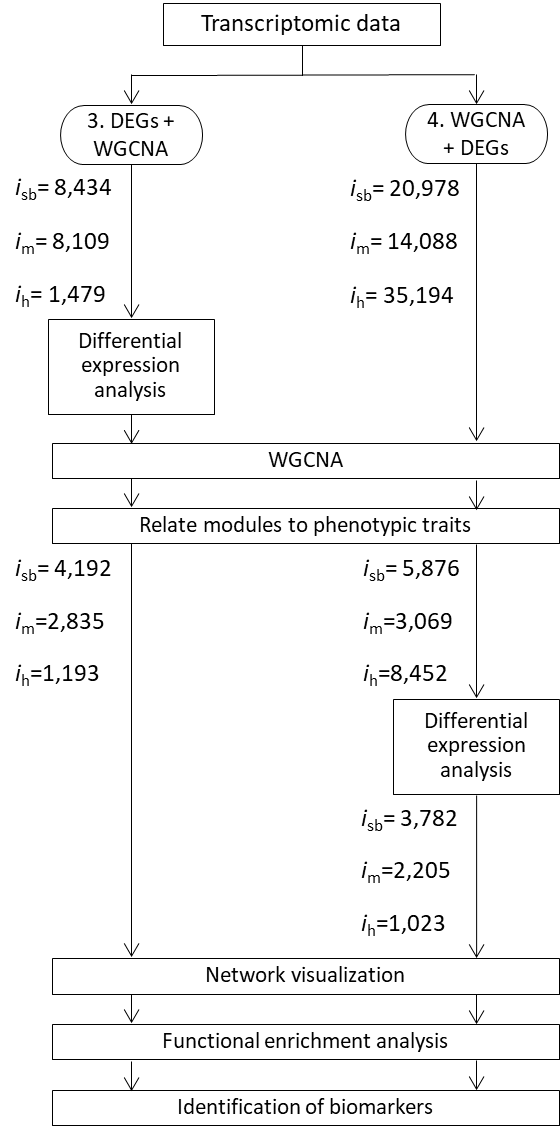
**

**Supplementary figure 2.** Flow diagram and number of genes of methods #3 and #4. Flow diagram and number of genes included at each step of the two different approaches compared in the present study, #3 and #4, in the sea bass (*i*_sb_), mouse (*i*_m_), and human (*i*_h_), where “*i”* stands for the number of genes kept for downstream analysis.

*Mus musculus*


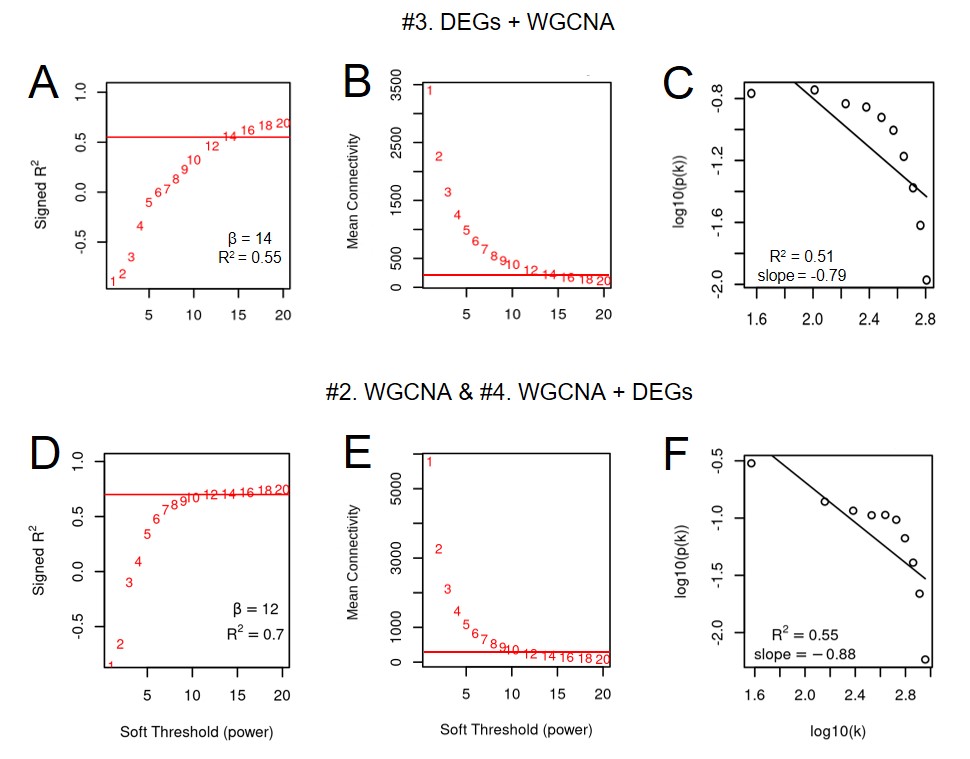


**Supplementary figure 3.** Determination of soft-threshold power in the WGCNA using the mouse gonadal transcriptome. Soft-thresholding power analysis was used to obtain the scale-free fit index (ranging from 1 to 20) of network topology, for method #3 (**A**) and #4 (**D**). Mean connectivity when using method #3 (**B**) and method #4 (**E**). Scale-free topology when using the selected soft threshold in (**C**) method #3 (β = 14) and (**F)** method #4 (β = 12). The x-axis shows the logarithm of whole network connectivity, and the y-axis the logarithm of the corresponding frequency distribution. Scale-free topology is represented by the distribution of this plot showing a straight line, approximately.


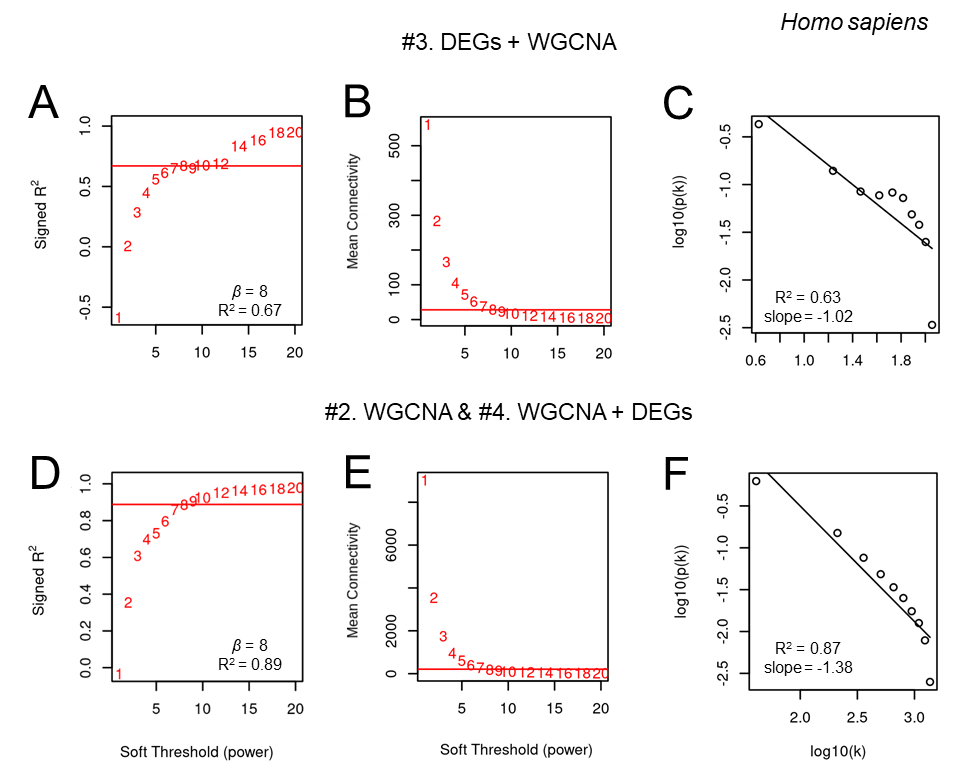


**Supplementary figure 4.** Determination of soft-threshold power in the WGCNA using the human gonadal transcriptome. Soft-thresholding power analysis was used to obtain the scale-free fit index (ranging from 1 to 20) of network topology, for method #3 (**A**) and #4 (**D**). Mean connectivity when using method #3 (**B**) and method #4 (**E**). Scale-free topology when using the selected soft threshold in (**C**) method #3 (β = 8) and (**F)** method #4 (β = 8). The x-axis shows the logarithm of whole network connectivity, and the y-axis the logarithm of the corresponding frequency distribution. Scale-free topology is represented by the distribution of this plot showing a straight line approximately.

**
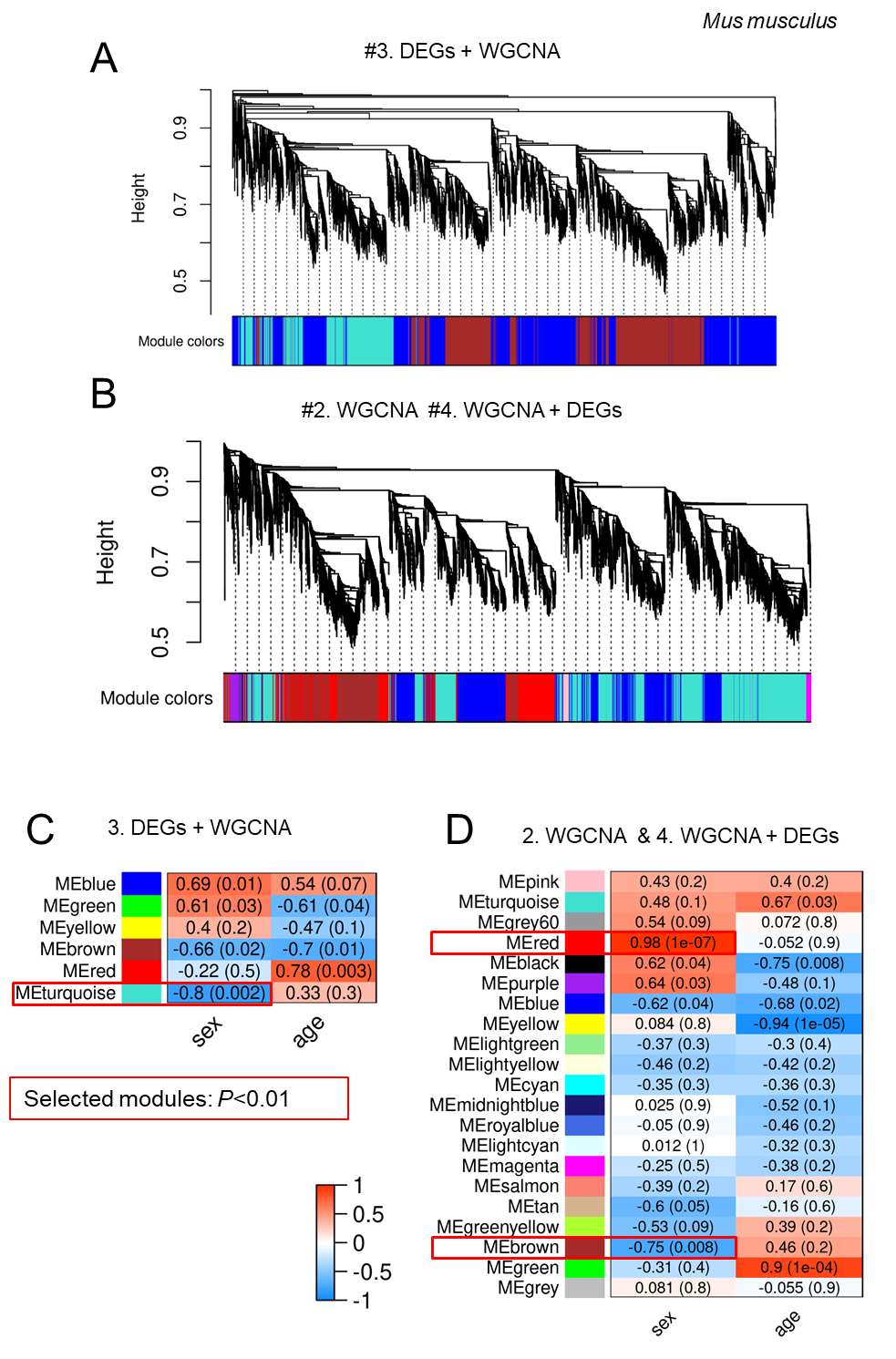
**

**Supplementary figure 5.** Identification of gene modules associated with sex in the mouse. Gene hierarchical cluster analysis using method #3 (**A**) and #4 (**B**). Each color represents a module in the constructed gene co-expression network by WGCNA. Heat maps of the correlation between sex with module eigengene distances in mouse dataset using **C**) method #3 and **D**) method #4. Each module has a color assigned shown in the side bar. The heat map is colored from red (1, positive) to blue (-1, negative) to indicate the level of correlation with the trait of interest. The red boxes highlight the selected modules for further analysis associated with sex (*P* < 0.01).

**
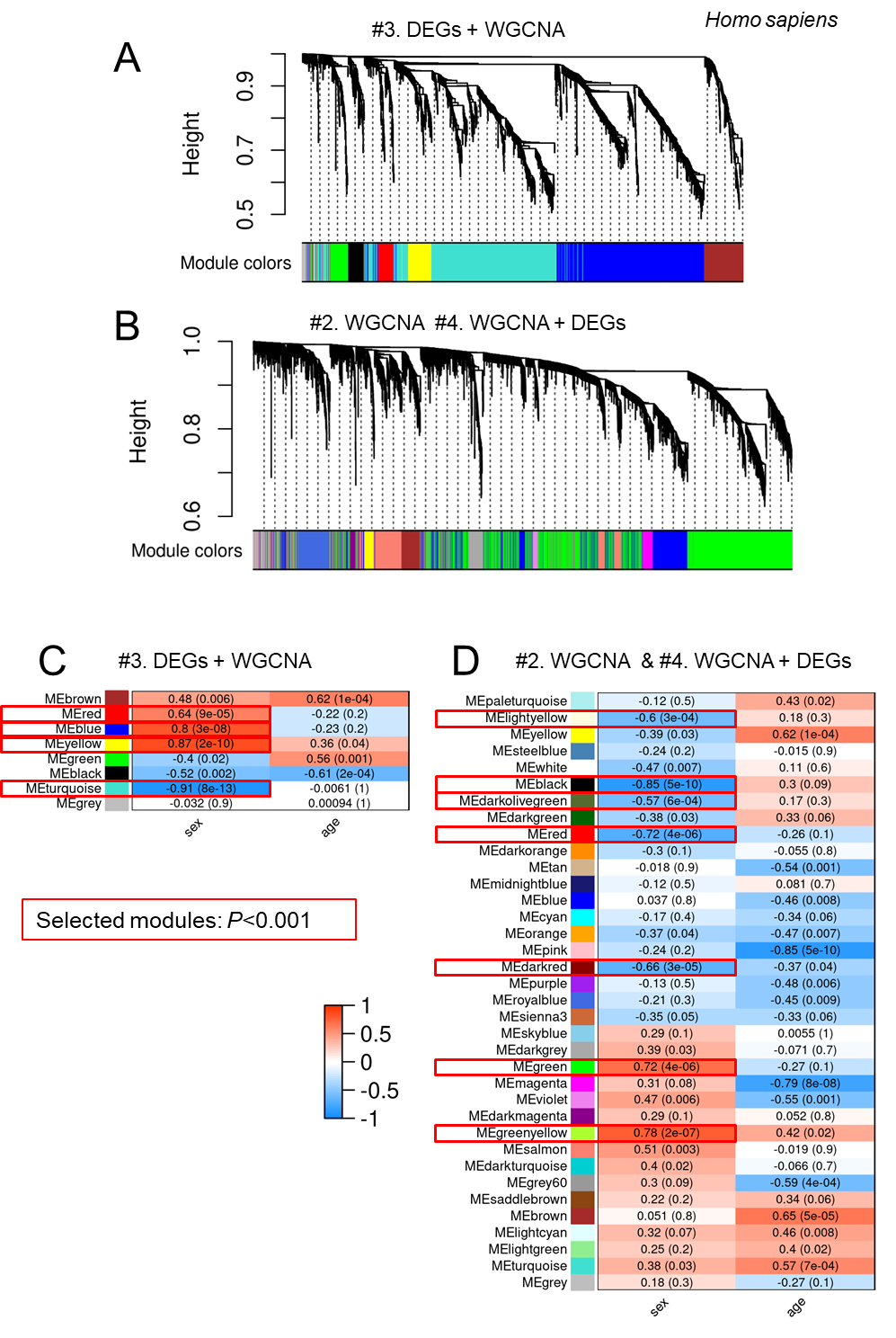
**

**Supplementary figure 6.** Identification of gene modules associated with sex in the human. Gene hierarchical cluster analysis using method #3 (**A**) and #4 (**B**). Heat maps of the correlation between sex with module eigengene distances in human dataset using **C**) method #3 and **D**) method #4. Each module has a color assigned shown in the side bar. The heat map is colored from red (1, positive) to blue (-1, negative) to indicate the level of correlation with the trait of interest. Red boxes highlight the selected modules for further analysis associated with sex (*P* < 0.001).

**
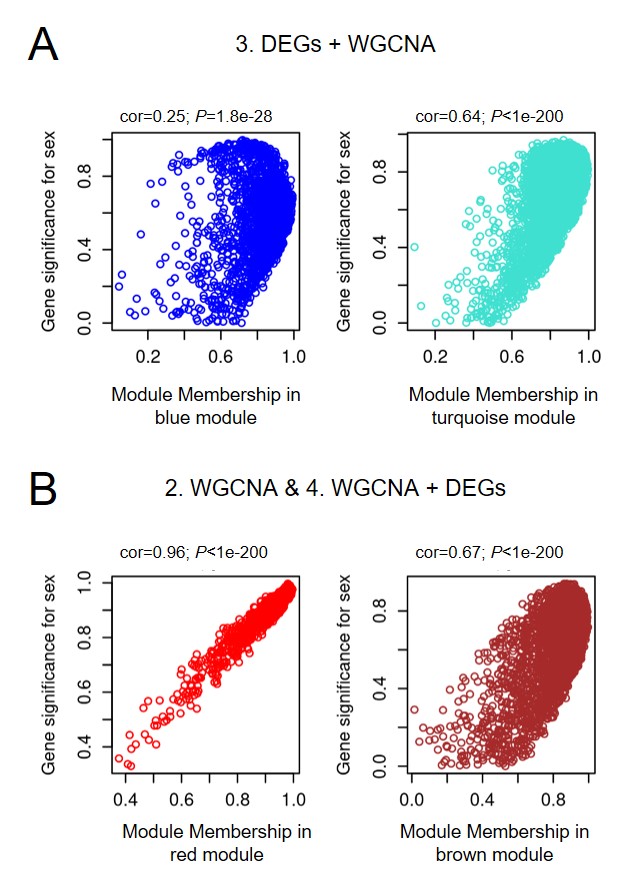
**

*Mus musculus*

**Supplementary figure 7.** Filtering of genes based on network properties in the mouse. Scatterplots of correlation between Gene Significance (GS) vs module membership of each module which were used to determination the interesting modules for sex trait in the gonadal transcriptome of mouse using method #3 (**A**) and #2 and/or #4 (**B**). The modules were selected when GS was positively and significantly correlated to modular membership.


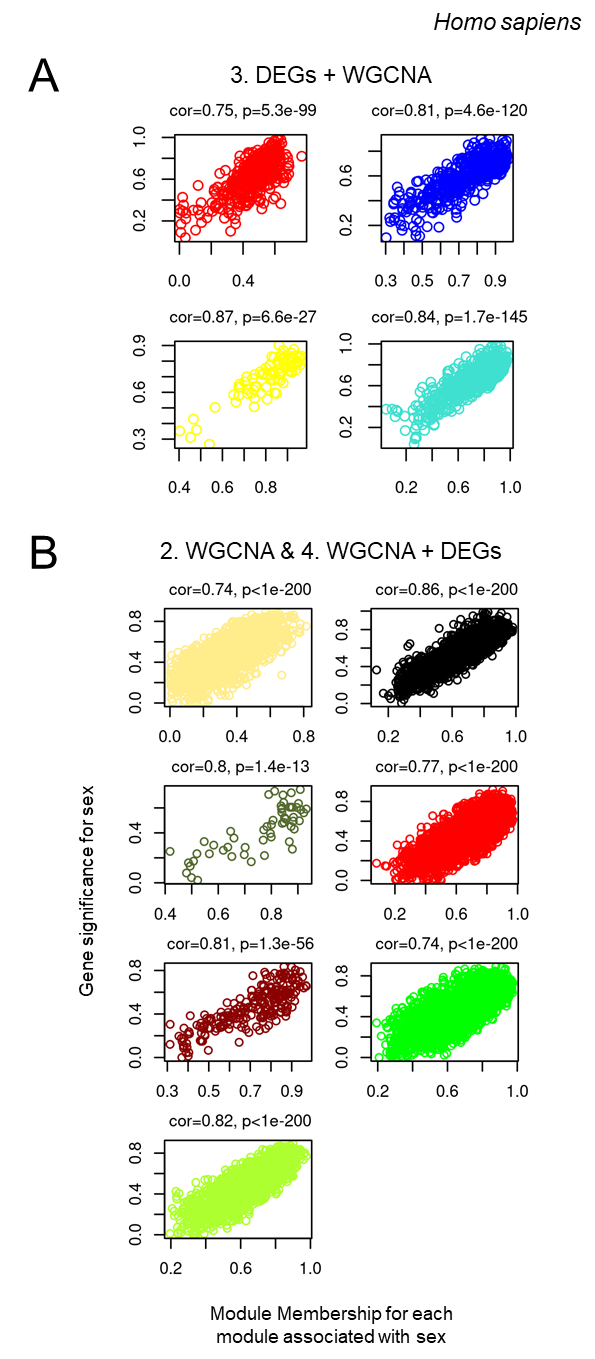


**Supplementary figure 8.** Filtering of genes based on network properties in the human. Scatterplots of correlation between GS vs module membership of each module which were used to determination the interesting modules for sex trait in the gonadal transcriptome of human using method #3 (**A**) and #2 and/or #4 (**B**). The modules were selected when GS was positively and significantly correlated to modular membership.

**
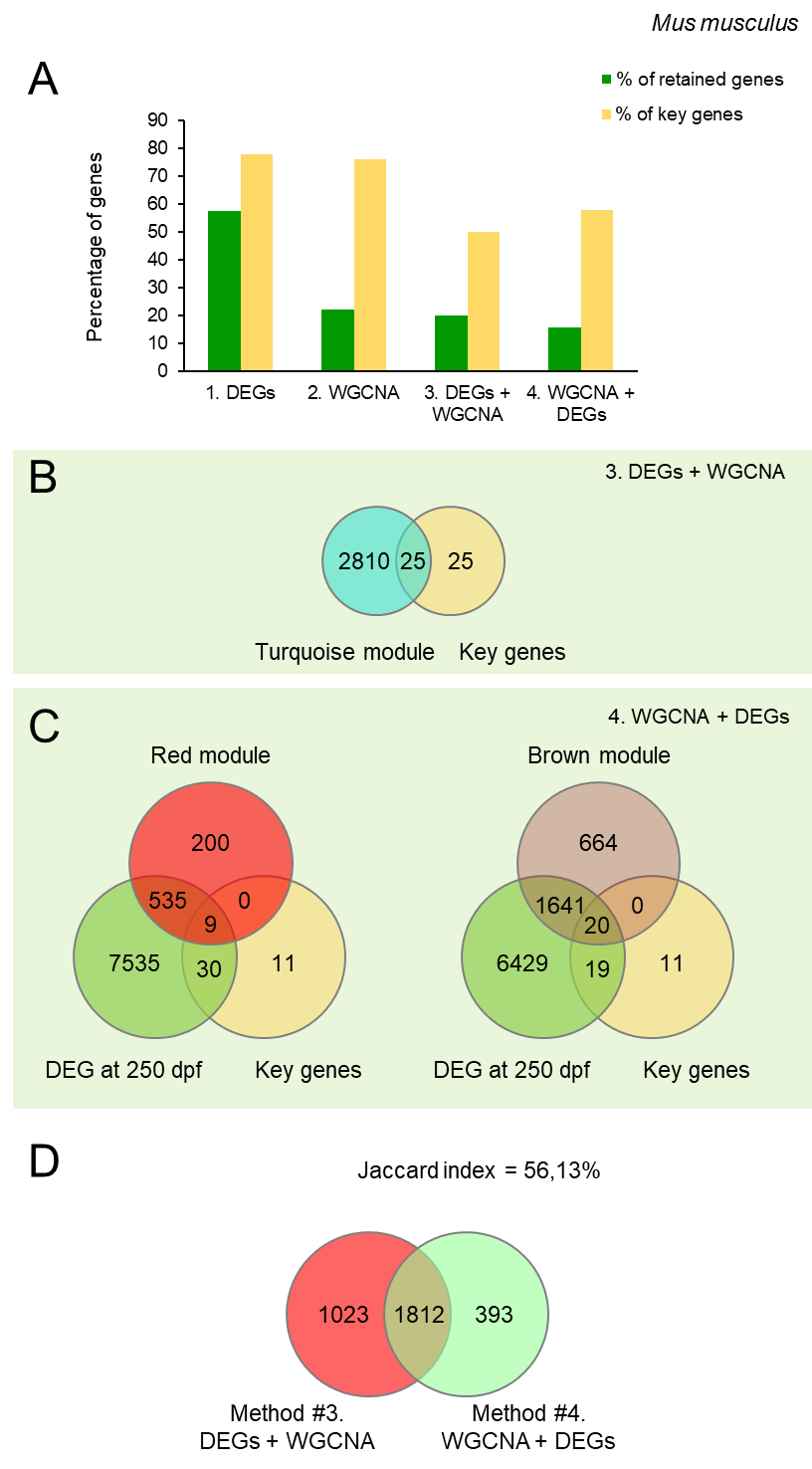
**

**Supplementary figure 9.** Identification of key genes involved in sex differentiation of mouse. **A.** Percentage of genes and key genes retained according to the methods mentioned in this study. Venn diagram of the key genes found in each module using method #3 (**B**) and #4 (**C**). **D.** Venn diagram of the genes retained using method #3 vs method #4 with the Jaccard index value showing low similarity between the genes retained by each method.

**
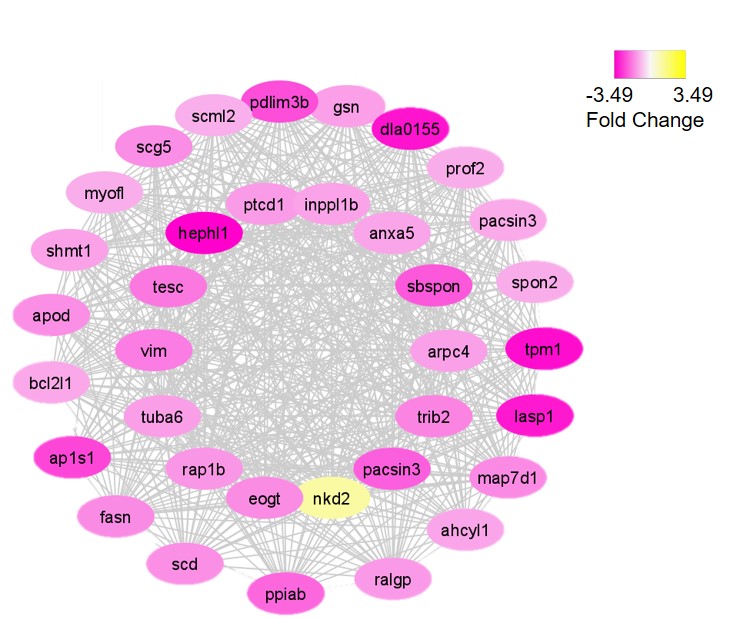
**

**Supplementary figure 10.** DEGs co-expression network of the magenta module using method #4 in sea bass data. Downregulated genes in females are shown in magenta and upregulated genes are indicated in yellow. The position of the nodes or genes indicates the degree range of each gene, which is higher to lower from inside to outside of the net.

**
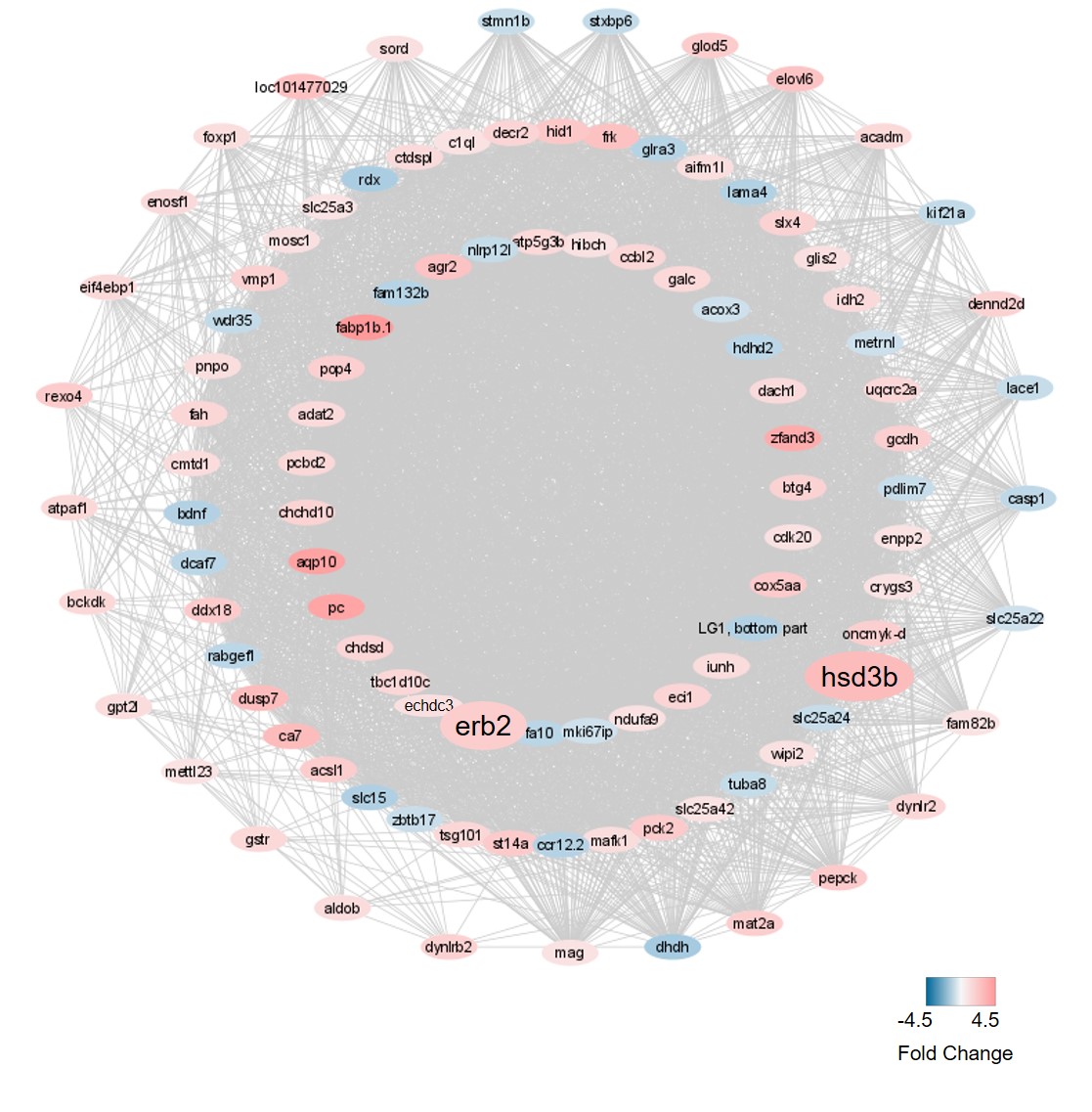
**

**Supplementary figure 11.** DEGs co-expression network of the pink module using method #4 in sea bass data. Downregulated genes in females are shown in blue and upregulated genes are indicated in pink. The position of the nodes or genes indicates the degree range of each gene, which is higher to lower from inside to outside of the net. Key genes were shown in bigger nodes.

**
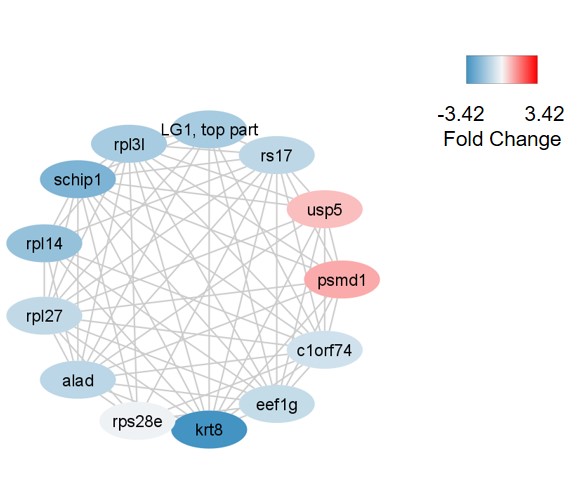
**

**Supplementary figure 12.** DEGs co-expression network of the sky blue module using method #4 in the sea bass data. Downregulated genes in females are shown in blue and upregulated genes are indicated in red.
